# Regulation of dynamic spatiotemporal inflammation by nanomaterials in spinal cord injury

**DOI:** 10.1186/s12951-024-03037-8

**Published:** 2024-12-19

**Authors:** Zeping Liu, Chunyu Xiang, Xu Zhao, Toshimi Aizawa, Renrui Niu, Jianhui Zhao, Fengshuo Guo, Yueying Li, Wenqi Luo, Wanguo Liu, Rui Gu

**Affiliations:** 1 Department of Orthopedic Surgery, China-Japan Union Hospital of Jilin University, Changchun, 130033 PR China; 2 Department of Orthopedics, Third Military Medical University, Xinqiao Hosp, 83 Xinqiao Main St, Chongqing, 400037 PR China; 3 Department of Orthopedic Surgery, Tohoku University Graduate School of Medicine, Sendai, 980-8574 Japan; 4 Department of Hand & Foot Surgery, China-Japan Union Hospital of Jilin University, Changchun, 130033 PR China

**Keywords:** Spinal cord injury, Nanomaterials, Inflammation, Spatiotemporal dynamic, Astrocyte, Microglia

## Abstract

Spinal cord injury (SCI) is a common clinical condition of the central nervous system that can lead to sensory and motor impairment below the injury level or permanent loss of function in severe cases. Dynamic spatiotemporal neuroinflammation is vital to neurological recovery, which is collectively constituted by the dynamic changes in a series of inflammatory cells, including microglia, neutrophils, and astrocytes, among others. Immunomodulatory nanomaterials can readily improve the therapeutic effects and simultaneously overcome various drawbacks associated with treatment, such as the off-target side effects and loss of bioactivity of immune agents during circulation. In this review, we discuss the role of dynamic spatiotemporal inflammation in secondary injuries after SCI, elaborate on the mechanism of action and effect of existing nanomaterials in treating SCI, and summarize the mechanism(s) whereby they regulate inflammation. Finally, the challenges and prospects associated with using nanotechnology to modulate immunotherapy are discussed to provide new insights for future treatment. Deciphering the intricate spatiotemporal mechanisms of neuroinflammation in SCI requires further in-depth studies. Therefore, SCI continues to represent a formidable challenge.

**Graphical abstract:**

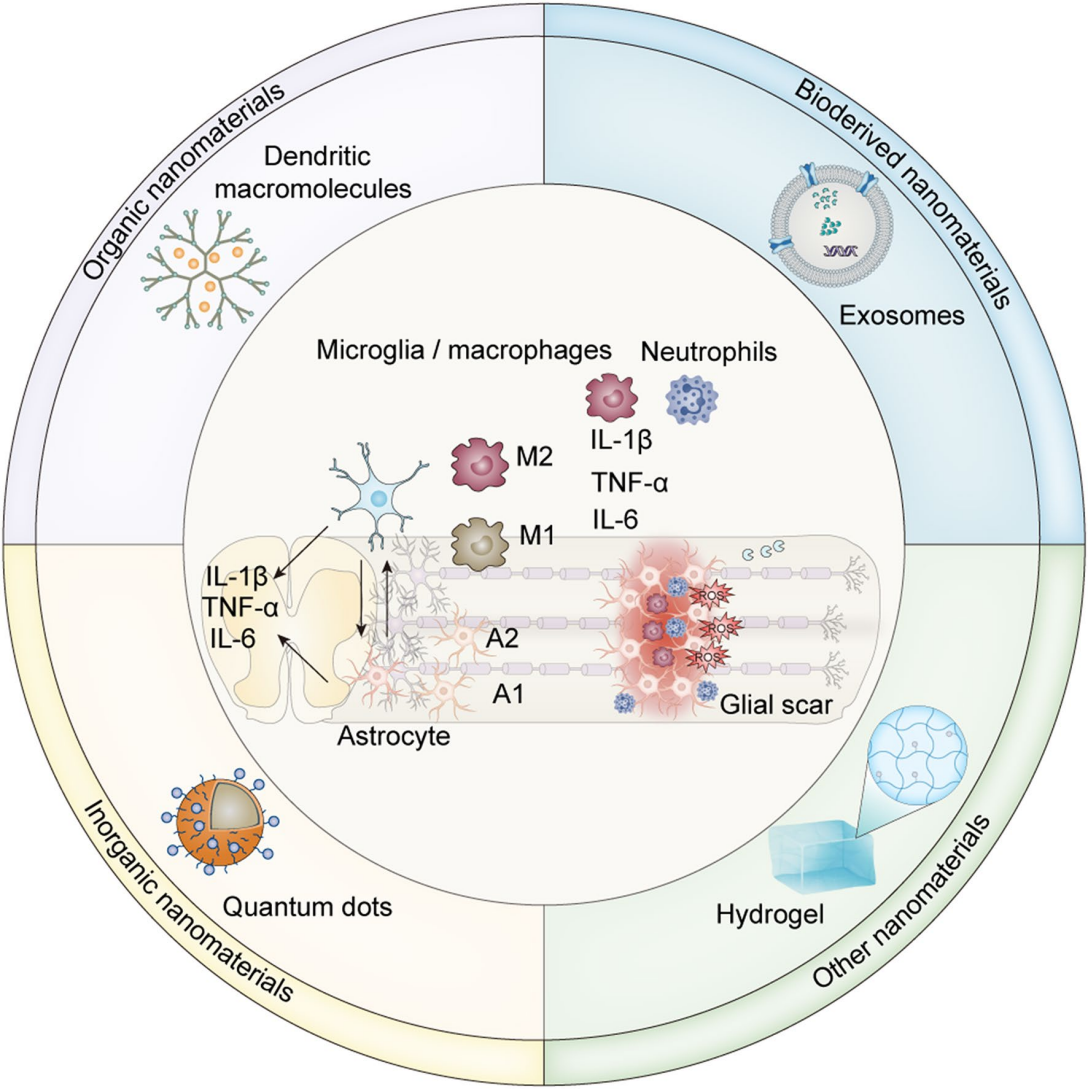

## Introduction

Spinal cord injury (SCI) is a type of central nervous system (CNS) lesion caused by trauma, inflammation, tumors, or other factors, which can impair sensory, motor, and autonomic nervous functions to various degrees (below the injury level) [1, 2]. With the progression of SCI, patients may also develop upward neurological deficits and impaired brain function [3, 4]. In severe cases, sensory and motor functions can be lost, markedly impacting the quality of life and imposing a substantial burden on patients and society [5]. Moreover, SCI treatment can impose a substantial financial burden, with more than US $3 billion spent annually in the United States on SCI management. Globally, approximately 250,000 to 500,000 new cases of SCI are diagnosed each year, with trauma representing the primary cause. In particular, motor vehicle collisions account for ~ 38.2% of the SCI cases globally, followed by falls (32.3%) [6]. Among all injury sites, the incidence of cervical SCI is considerably higher than that of chest and waist SCI, accounting for more than 50% of the traumatic SCI cases [7]. 

There are two predominant types of SCI—primary and secondary injury [8]. Primary injury is often directly caused by violent trauma, whereas secondary injury is characterized by a complex cascade of amplified response processes, including inflammatory reactions, elevated levels of reactive oxygen species (ROS), glutamate toxicity, calcium overload, ischemia, apoptosis, and edema, ultimately resulting in neurological dysfunction [9]. Spatiotemporal dynamic neuroinflammation plays an important role in secondary injury [10–12]. Inflammatory cells play different roles in inflammatory activation, inflammatory abatement, tissue repair, and homeostatic remodeling; inflammatory mediators such as cytokines and chemokines exert unique effects at different stages of inflammation. For example, early inflammatory responses can help clear tissue debris and stimulate neuronal regeneration [13, 14]. As inflammation progresses, numerous cytokines, proteases, and ROS are released, causing further damage [15]. Additionally, multiple changes are observed in the counts and functions of various cell subsets, while the cumulative effect of multiple inflammatory cells induces inflammation following SCI. These spatiotemporal dynamic neuroinflammatory processes directly damage the nerve tissue and inhibit nerve regeneration; these effects are further buttressed by the poor post-injury microenvironment [16]. 

During the dynamic progression of inflammation, the transition from beneficial to harmful inflammation poses substantial challenges and negatively impacts neurological recovery. The existing treatments for SCI primarily include surgery and methylprednisolone (MP) therapy; however, their disadvantages are becoming increasingly prominent. Although the spinal stability of patients often recovers after surgery, the associated nerve damage can be irreversible, preventing full recovery of function [5]. Moreover, MP treatment may be complicated by massive digestive tract hemorrhage, pulmonary embolism, arrhythmia, sepsis, and other issues [17]. Therefore, an urgent need exists to develop more effective treatments [17, 18]. 

**Scheme 1:**
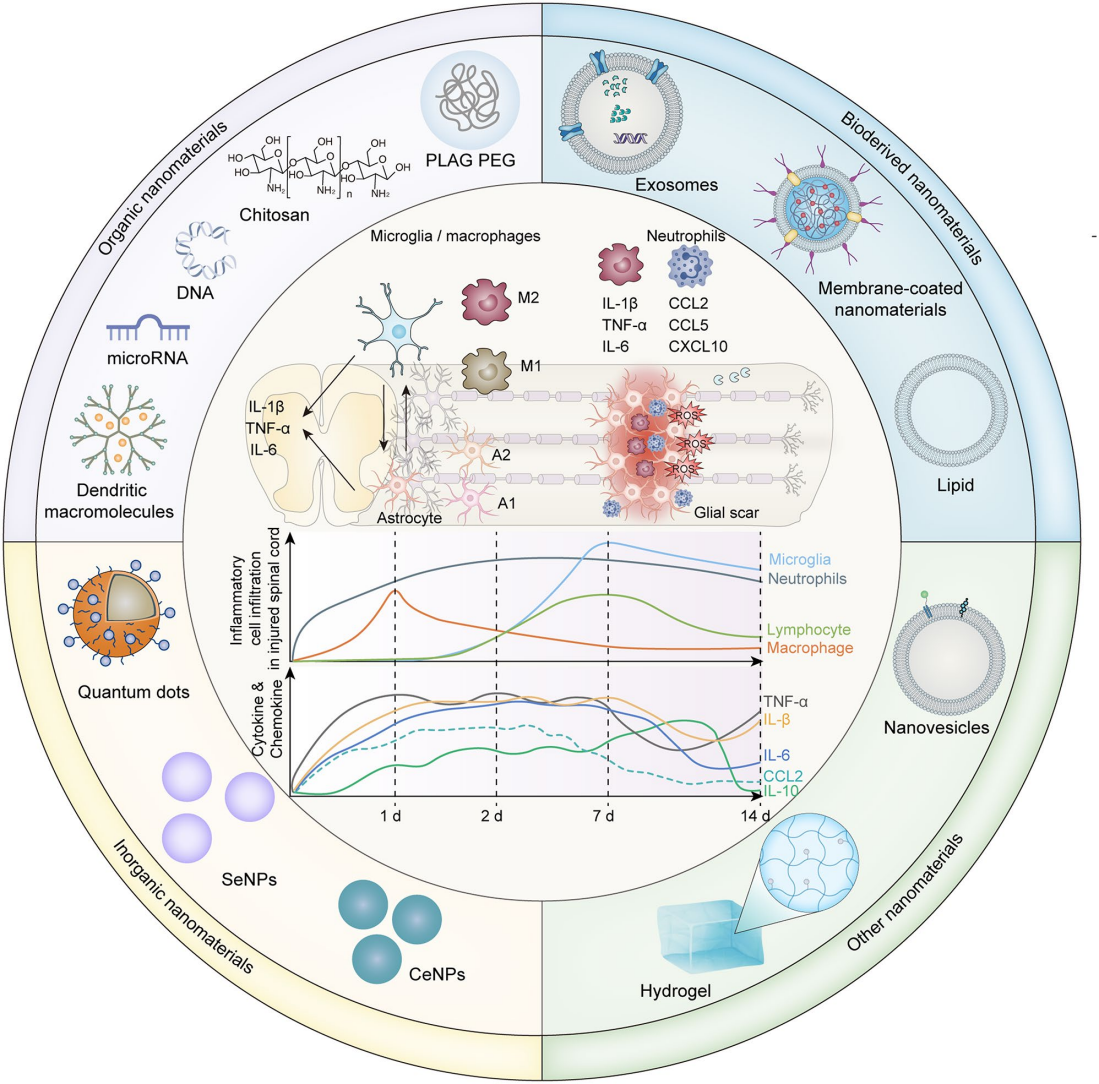
A schematic depiction of the inflammatory responses mediated by immune cells and cytokines, their dynamic shifts in the context of traumatic spinal cord injury, and the classification and structure of nanomaterials used for inflammation modulation

As an emerging therapeutic modality, nanomaterials are favored in various fields owing to their unique properties, i.e., small size, good targeting, and slow or stepwise release of drugs. These properties can also add to the unique advantages of nanomaterials in the dynamic treatment of SCI inflammation. This article discusses the current status and latest progress in the management of inflammation in SCI and the development of treatments targeting the inflammatory microenvironment after SCI (Scheme 1). The objective of this article is to lay the groundwork for further development of various nanomaterials aimed at treating inflammation.

## Overview of SCI pathophysiology

**Fig. 1:**
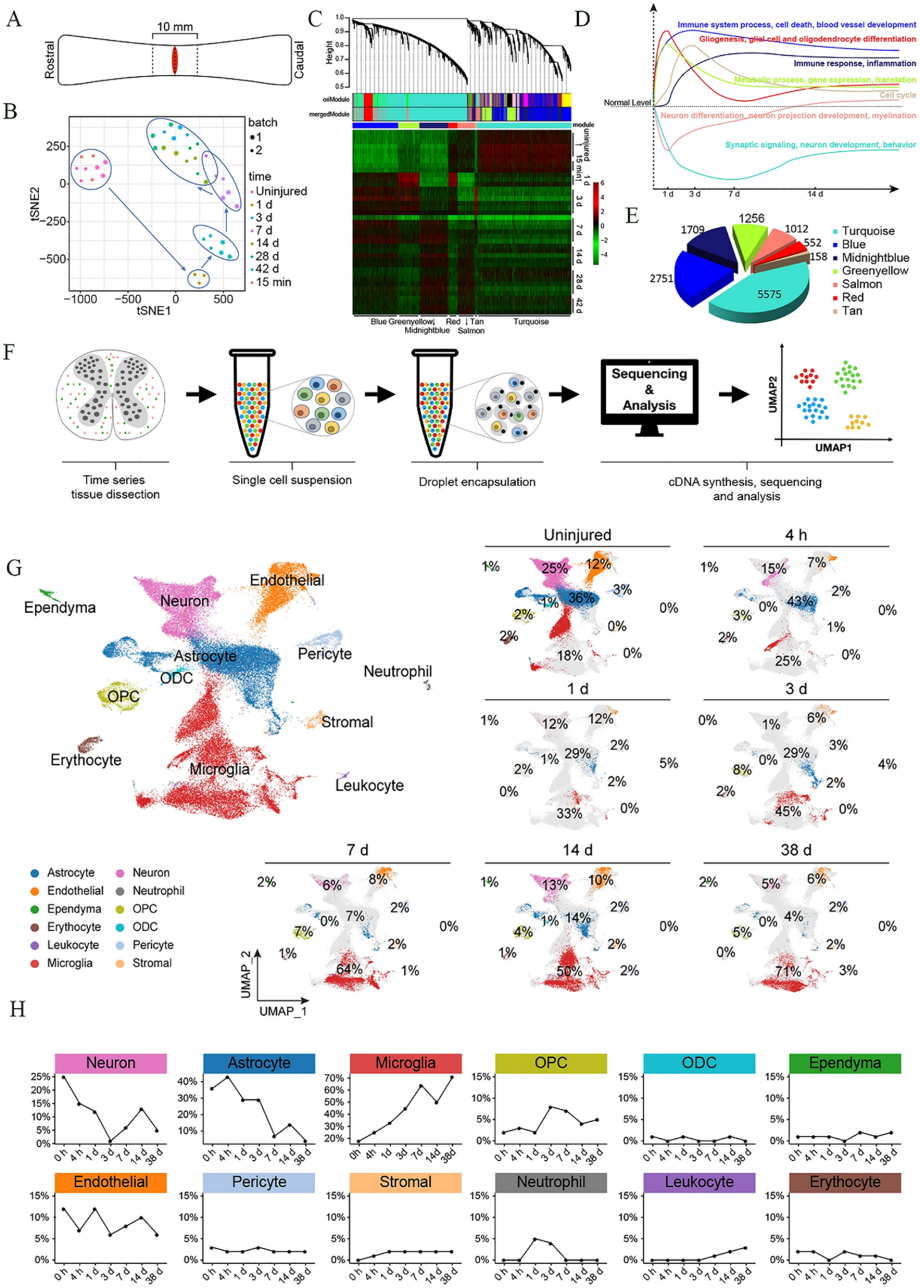
Temporal transcriptomic changes post-spinal cord injury (SCI), as revealed by population-based (bulk-RNA-seq) and single-cell RNA sequencing (scRNA-seq): **A**) Schematic representation of the tissue sampling process for bulk-RNA-seq and scRNA-seq. **B**) The t-distributed Stochastic Neighbor Embedding (t-SNE) plot demonstrates consistent sequencing outcomes across different samples. **C**) Hierarchical clustering of 39 samples showing the expression heatmap of each module across all samples. **D**) Gene ontology (GO) terms for seven gene modules and their average gene expression changes over time post-SCI. **E**) Pie chart illustrating the number of genes within each module. **F**) Overview of the 10× Genomics scRNA-seq experimental workflow. **G**) Visualization of spinal cord cells from different samples using Uniform Manifold Approximation and Projection (UMAP). **H**) Line graph depicting the temporal changes in the relative proportions of 12 major cell types identified through scRNA-seq. Used under the terms of the Creative Commons Attribution 4.0 International License (CC BY 4.0). License details available at https://creativecommons.org/licenses/by/4.0/”

SCI include two types, i.e., primary and secondary injury [8]. Primary injury refers to the momentary effect of a mechanical force on the spinal cord [19]. At this stage, there may be compression or contusion of the spinal cord, damage to the axons and neurons [20], reduced and interrupted spinal cord blood flow, increased blood–spinal barrier permeability, and bleeding at the site of injury [21, 22]. Meanwhile, secondary injury may result in gradual enlargement of the necrotic area, aggravation of the destruction and loss of function of the neuronal area, and impairment of neuronal regeneration [23]. Causes of secondary injury include inflammatory responses, elevated levels of ROS, calcium overload, ischemia, edema, excitatory toxicity of glutamate, and the onset of necrosis or apoptosis, leading to neurological loss [9]. The cellular and molecular changes associated with secondary injury exhibit dynamic spatiotemporal characteristics. Li et al. observed pathological changes in 12 key cell types, with three of these infiltrating the spinal cord at specific times post-injury (Fig. 1A-F) [24]. The most significant alterations occurred three days post-injury (dpi), and by 14 dpi, a second wave of microglial activation became apparent (Fig. 1G-H). A comprehensive understanding of the processes could inform the development of treatment strategies that target distinct disease processes at the most effective times.

Recently, a study showed that neuroinflammation is vital in secondary injury [25]. Inflammation is caused by the activation of resident immune cells (microglia and astrocytes) and the recruitment of peripheral immune cells (macrophages and neutrophils). Microglia can improve the anti-inflammatory microenvironment at the injury site and plays important roles in various neurological diseases. Reactive microglia recruit circulating white blood cells, including neutrophils, macrophages, T lymphocytes, and B lymphocytes, to the injury site. In addition to damaging the neurons, the recruited white blood cells can clear tissue debris and support nerve regeneration. Following infiltration, neutrophils recruit other immune cells to clear cell debris, produce inflammatory factors, and promote tissue healing. For instance, astrocytes respond to pathological conditions in the CNS and work with neutrophils to isolate the damaged site and rebuild the blood–brain barrier. The inflammatory factors secreted by astrocytes activate the microglia, while the chemokines secreted by astrocytes induce inflammatory cell infiltration, leading to increased vascular permeability, leukocyte infiltration, glial cell activation, and increased production of inflammatory mediators such as cytokines and chemokines, gradually exacerbating inflammation [26]. Under microglial induction, astrocytes can amplify the inflammatory effect and cause neurotoxicity, leading to cell death and scar formation.

In the course of inflammatory response, numerous inflammatory cells, such as neutrophils and macrophages, release pro-inflammatory cytokines, chemokines, ROS, and proteolytic enzymes, causing an immune response [27]. Numerous cytokines produced during inflammatory reactions, including tumor necrosis factor (TNF)-α and interleukin (IL)-6, can diffuse to other organs, causing systemic inflammatory changes [28]. In the first few hours, microglia/macrophages, astrocytes, and neurons secrete the pro-inflammatory cytokines IL-1β, TNF-α, and IL-6 [29]. Reactive astrocytes can also release C-C Motif Chemokine Ligand 2(CCL2), CCL3, CCL5, and nitric oxide (NO), which upregulate IL-1 α, IL-1 β, IL-6, and TNF-α and downregulate macrophage migration inhibitory factor (MIF). Although some of these inflammatory factors can help maintain a good microenvironment and reduce tissue damage, most stimulate inflammatory cells and disrupt the blood–brain barrier, thereby exacerbating local inflammation [30]. 

Ultimately, SCI damages the endothelial cell sheath and blood vessels, leading to neuronal death. Similarly, inflammatory reactions can lead to iron deposition, resulting in programmed cell death, which gradually increases inflammation [31, 32]. With the progression of inflammation, inflammatory cells release cytokines, proteases, ROS, and other factors [33], which hinder the regeneration of axons and neurons, promote glial scarring, and cause more serious injury [34–36]. The development of fibrotic scars following tissue damage is a critical phase in the healing process, yet it paradoxically limits the beneficial aspects of the inflammatory response and impedes axon regeneration. This occurs as the dense extracellular matrix laid down by fibroblasts and other cells creates a formidable barrier [33]. Consequently, the persistent presence of such scars contributes to ongoing dysfunction by impeding reparative mechanisms and disrupting normal tissue architecture [37]. Axonal growth is also limited by the presence of related growth inhibitors and reduced expression of related genes [38]. After SCI, mitochondrial function is impaired, and many free radicals are produced, aggravating the injury [39]. Inflammation can also cause systemic pathological and organ function changes, e.g., changes in vascular permeability [40, 41]. Recent studies have shown that the spatiotemporal progression of inflammation from the acute to the chronic phase following SCI is a complex process involving myriad cells, inflammatory factors, and chemokines [42]. This process has a significant impact on neurological function and recovery, with the acute phase characterized by immediate inflammatory cell infiltration, chemokine and cytokine release, and blood-spinal cord barrier (BSCB) disruption, leading to edema formation, neuronal damage, and axonal degeneration; conversely, the chronic phase is characterized by persistent inflammation, a shift toward anti-inflammatory mediators, gliosis, ongoing neurodegeneration, and potential neuroplasticity [43]. 

## Dynamic spatiotemporal inflammatory responses in SCI

**Fig. 2:**
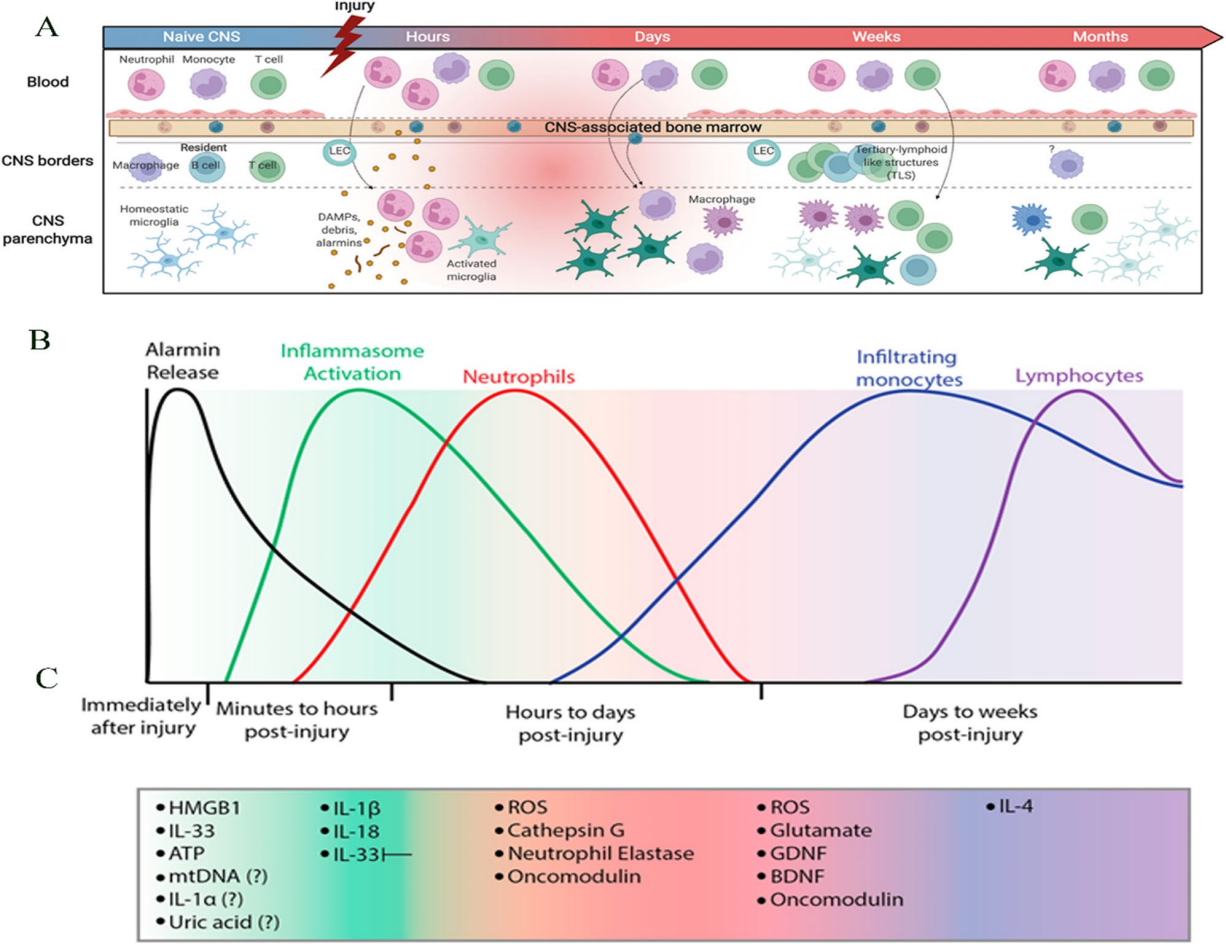
Inflammation associated with central nervous system (CNS) injury. **A**) Compartmentalization of the immune response after CNS injury. **B**) Phases of molecular and cellular inflammation after CNS injury. **C**) Activity of specific inflammatory molecules at different time points corresponding to the injury. Reproduced from with permission [54, 55]. Licensed under CC BY 4.0. Copyright © 2023 Elsevier

After SCI, inflammation is thought to spread centripetally, i.e., it moves from the periphery to the center [44]. A series of inflammatory cells infiltrate the regions around the spinal cord [45], and concurrently secrete inflammatory factors (IL-1β, IL-6, TNF-α, and IFN-γ) and chemokines (CCL2, CCL3, CCL5, CCL20, C-X-C Motif Chemokine Ligand 10 (CXCL10), and CXCL12), aggravating the inflammatory response and inhibiting axonal regeneration, thereby leading to the loss of nerve function [46–50]. The secreted inflammatory cytokines and chemokines elevate immune cell infiltration, further promoting inflammatory cytokine production and ROS, NO, and chemokine release [51, 52]. Early inflammation involves four types of immune cells (Fig. 2A). (1) Microglia are the first inflammatory cells to reach the site of injury. (2) Neutrophils follow closely, peaking 24 h post-injury (hpi). Neutrophils can engulf and clear debris; secrete proteases, elastases, and myeloperoxidase; and release ROS. (3) Circulating monocytes/macrophages are subsequently recruited and they release various cytokines and perform phagocytosis. (4) Lymphocytes progressively invade the lesion site where, together with macrophages, they secrete cytokines [53]. These inflammatory cells further recruit infiltrating inflammatory cells. Therefore, with progressive inflammation, various inflammatory cells and cytokines exhibit spatiotemporal dynamism (Fig. 2B-C) [42]. Understanding such dynamic processes is critical for designing effective treatment targets in the context of SCI.

Microglia are the first to respond by extending protrusions toward the lesion site (within seconds to minutes); they then transform into amoeba-like phagocytes, engulfing cells and clearing debris, including myelin sheath fragments [56]. These cells express CD11b and secrete anti-inflammatory factors, pro-inflammatory factors, prostaglandins, cytokines, and ROS [57, 58]. Moreover, under conditions of microglial activation and disruption of the BSCB, blood-derived immune cells, such as neutrophils, monocytes/macrophages, and lymphocytes, can infiltrate the spinal cord tissue [59]. Neutrophils are the second immune cell type to reach the site of injury, first arriving ~ 4 h after CNS injury, peaking in abundance 24 hpi, and persisting at the site of injury for 2–7 days [60]. These cells can sustainably release pro-inflammatory cytokines, oxidases, ROS, chemokines, lyases, and other molecules, continuously exacerbating the inflammatory response [61, 62]. CD3 + lymphocytes become evident in the spinal cord only after 3 dpi, peak at 7 days, and sustained T-cell response is observed up to 180 days [63]. However, in the context of SCI, T cells have been identified as early as 1 dpi, peaking at 7 days. Sustained T-cell responses have been observed up to 180 dpi. Cytotoxic CD8 + CD28 + T cells dominate during the first two weeks following SCI, during which the survival time of cells can be extended, and the proportion of regulatory CD8 + T cells can increase [63]. 

**Fig. 3:**
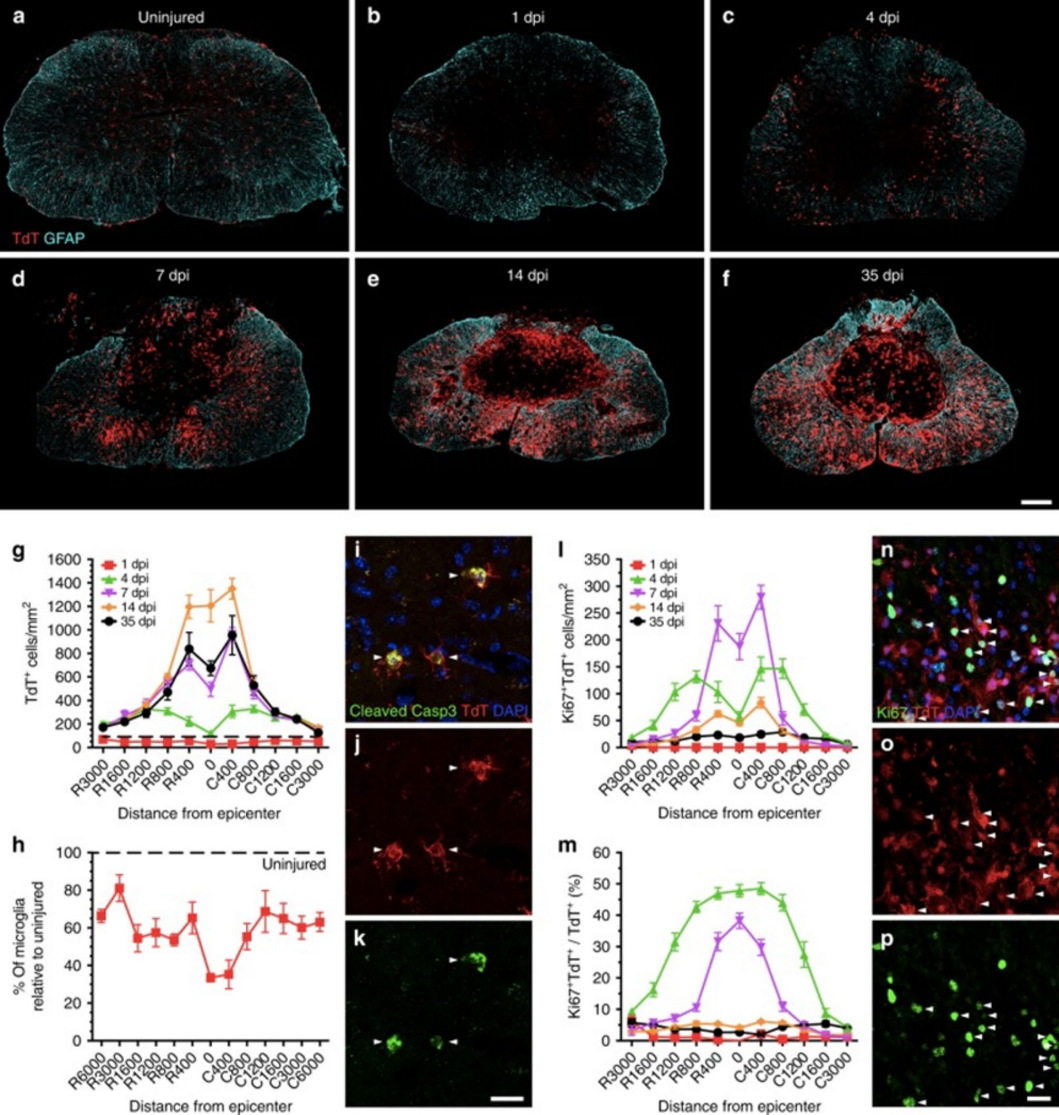
After spinal cord injury, microglia extensively proliferate and accumulate at the injury boundary. **A**–**F**) Confocal microscopic images displaying representative spinal cord sections from uninjured to 1–35 dpi. **G**–**P**) Number of microglia in each group and the expression of apoptosis and proliferation markers. Reproduced with permission [72]. Licensed under CC BY 4.0

In SCI, macrophages are the key phagocytic cells present at 3 dpi [64]. Infiltrating macrophages present in the peripheral circulation begin to infiltrate the spinal cord 1–3 dpi and enter the site of injury 3–4 dpi [24]. However, microglia and macrophages share several immunohistochemical markers, making it difficult to distinguish them in terms of morphology; hence, they are commonly referred to as microglia/macrophages [65]. The innate immune response triggered by microglia is augmented by peripheral myeloid cells, mainly neutrophils and monocytes, which migrate to the injury site [66]. Microglia/macrophage abundance peaks at 7 dpi [67]. Fourteen days after injury, the second wave of microglial/macrophage activation occurs, causing them to occupy the core area of the injury (Fig. 3A-P) [68]. Therefore, the initial 7 dpi are highly dynamic and offer a therapeutic window for intervention in spinal cord injuries. Microglia/macrophages can continue to aggregate within the core area of the injury for up to 28 dpi, where they can disrupt the BSCB continuously, leading to the continuous progression of inflammation [69]. After 14 dpi, cellular inflammation is prolonged for 180 days; at 60 dpi, another peak in microglia/macrophages is observed [70]. In fact, foam-like macrophages can be detected for up to one year after SCI [71]. 

The mechanism via which microglia/macrophages affect SCI may depend on their spatiotemporal activation [73]. In early-stage SCI, microglia/macrophages phagocytose circulating white blood cells, clear cell debris, and protect nerves [74]. As the injury worsens, microglia/macrophages secrete pro-inflammatory molecules, causing neuroinflammation and leading to secondary injury [75, 76]. The function of microglia/macrophages is determined by their phenotype, which evolves in response to the surrounding microenvironment. The M1 macrophage phenotype tends to enhance the inflammatory response and exacerbate neuroinflammation, while the M2 phenotype has anti-inflammatory effects and promotes tissue repair. However, there may be additional subtypes of microglia present in the injured spinal cord. Indeed, eight clusters of microglial subpopulations have been identified in mice with SCI at the single-cell level, each with distinct characteristics [24]. The optimal timeframe for converting reactive microglia to their neuroprotective phenotypes might be the first week post-SCI. The transformation of phenotypes is dynamic and regulated by various factors within the injured microenvironment. Therefore, microglia/macrophages may represent significant therapeutic targets for the next generation of treatments for SCI [24, 44]. 

Liddelow et al. [77] demonstrated that the release of IL-1α, TNF-α, and complement component 1q by microglia triggers transcriptional changes in astrocytes. These altered astrocytes then secrete inflammatory factors (such as IL-1β, IL-6, TNF-α, IL-17, and ROS) and chemokines (including CCL2 and CXCL10), further exacerbating the inflammatory response [78, 79]. Astrocytes can be induced to differentiate into the A1 and A2 phenotypes when exposed to neuroinflammation and ischemia, respectively. [77] A1 astrocytes are characterized by the upregulation of various pro-inflammatory cytokines and complement cascade genes that are destructive to the synapses. Conversely, A2 astrocytes are characterized by the upregulation of various anti-inflammatory cytokines, neurotrophic factors, and platelet reactive proteins, all of which are beneficial in the repair and reconstruction of neural tissue.

Neuroinflammation is characterized by a series of cellular and molecular changes in the spinal cord, which are jointly affected by various cytokines and chemokines [80]. Between 30 min and 1 hpi, microglia and astrocytes secrete IL-1β, TNF-α, and IL-6, i.e., the main players in the early stages of injury [81]. IL-1β and IL-6 stimulate the production of inducible nitric oxide synthase (iNOS) in astrocytes, microglia, and macrophages [82]. The NO produced as a result of iNOS activity participates in microglia-dependent demyelination and neuronal apoptosis [83]. Meanwhile, reduced IL-1β levels can inhibit lesion development, promotion of axonal plasticity, and improvement of neurological prognoses [84, 85]. IL-1β, TNF-α, and IL-6 are significantly upregulated within 3–6 hpi, leading to further recruitment and proliferation of microglia, astrocytes, and surrounding immune cells [86]. The expression of IL-1β, TNF-α, and IL-6 by neutrophils peaks 12 hpi. However, SCI is also characterized by the early upregulation of high mobility group box 1 protein (HMGB1), whose release precedes those of TNF-α and IL-1β by monocytes/macrophages via the nuclear factor (NF)-κB signaling pathway [87]. Twenty-four hours after SCI, HMGB1 expression is increased significantly, causing an increase in microglial cell abundance and TNF-α release [88]. Therefore, TNF-α, IL-1β, and IL-6 levels continue to rise within the first 24 h. In the post-injury period (spanning 1–7 days), there is a gradual decline in the number of neutrophils, leading to gradual decreases in TNF-α, IL-6, and IL-1β levels between 24 h and 7 d. However, their levels remain elevated significantly compared to those in the uninjured tissue [89]. In mice, a second surge in the expression of TNF-α and IL-1β occurs on day 14 post-SCI, concurrent with the secondary activation of microglia, which persists until 28 dpi. In contrast, the levels of IL-6 in rats and mice decline to baseline levels (14 dpi onward) and remain stable thereafter.

Although anti-inflammatory cytokines can decrease the levels of pro-inflammatory cytokines, they are often lacking or present at minimal levels following SCI [90]. IL-4, IL-10, and IL-13 are secreted by microglia, astrocytes, and circulating and peripheral immune cells and act as anti-inflammatory cytokines that downregulate pro-inflammatory cytokines [91]. IL-10 activates microglia/macrophages and astrocytes and reduces the levels of IL-1β and iNOS [92]. These anti-inflammatory cytokines can be upregulated within 4 hpi, indicating early initiation of the anti-inflammatory process. However, most studies indicate that levels of IL-4, IL-10, and IL-13 rise initially but then decline and stabilize at baseline levels [92]. This transient increase does not halt the progression of inflammation, as the recruitment and proliferation of microglia and astrocytes, along with the influx of surrounding immune cells, continue to fuel the inflammatory process [93]. After an initial surge 4 h following the injury, levels of IL-4, IL-10, IL-13, and TGF-β are maintained at baseline for 12–24 h. A significant increase in IL-10 levels reportedly occurs at 3 dpi, and the levels remain elevated until 7 dpi. Meanwhile, IL-4 levels continue to increase at 14 dpi and begin to decrease at 28 dpi, whereas IL-10 levels do not change significantly during this period. The levels of IL-4 and IL-3 decrease gradually at 28 dpi. From the initial 4 hpi to day 28, the generated inflammatory and chemotactic factors exacerbate the infiltration of immune cells and stimulate their production of additional inflammatory mediators [94]. After SCI, the CNS drives the migration of neural stem cells (NSCs) along the gradient of soluble chemokines, such as soluble chemokine 1, matrix binding factor 2, and matrix cell-derived factor 1α. Gradient migration is used to supplement cells and maintain normal tissue function and structure in the CNS. Collectively, the above-mentioned cells and cytokines are characterized by spatiotemporal dynamism. Spatiotemporal dynamics are often regulated precisely owing to their essential functions. Remarkably, macrophages display a high degree of adaptability, akin to activated microglia. Consequently, advanced targeted and cell-specific techniques are vital for a better understanding of the specific functions of the immune cells in SCI and to effectively leverage their therapeutic benefits. In addition, this complex interplay creates a spatiotemporal pattern of inflammation that is crucial for recovery following SCI. Therefore, understanding the spatiotemporal distribution of the factors during post-SCI inflammation could inform targeted management of inflammation in SCI [79, 95]. 

SCI-induced inflammation is initiated by several signaling pathways, such as the NF-κB, TGF-β/TGFBR, PI3K/AKT, JAK/STAT3, and MAPK pathways [96–102]. The pathways of action are different under the application of various nanomaterials. The NF-κB pathway is the most common pathway modulating inflammation after nanomaterial use. Activation of the NF-κB signaling pathway is closely associated with the overexpression of pro-inflammatory cytokines following SCI. Specifically, activation of NF-κB leads to its translocation into the nucleus, where it promotes the transcription of genes encoding pro-inflammatory cytokines, such as TNF-α, IL-1β, and IL-6. These cytokines contribute to secondary damage and exacerbate the inflammatory environment, leading to further tissue damage and neuronal cell death. Various nanomaterials have been developed to target the pathways involved in SCI-induced inflammation. Zhang et al. reported that layered double hydroxide-coupled NT3 nanoparticles (NPs) downregulate the Piezo1/NF-κB pathways and exert anti-inflammatory effects [96]. Zhang et al. have developed a therapeutic nanofiber based on a self-assembling peptide that carries emodin (rhein) and promotes the transition of M1-type macrophages to M2-type macrophages by regulating the NF-κB/STAT3 signaling pathway [98]. In addition, Yang et al. have developed an injectable and bioadhesive gelatin-based hydrogel that delivers folate-functionalized polydopamine NPs (FA-PDA@Leon) to suppress inflammation by downregulating the JAK2/STAT3 signaling pathway in macrophages, in turn reducing ferritinophagy/ferroptosis [100]. Shen et al. developed a rapamycin (Rapa)-loaded hollow mesoporous Prussian blue (HMPB)-based nanozyme (RHPAzyme) that scavenges ROS and reduces inflammation by inhibiting the MAPK/AKT signaling pathway, thereby alleviating neuronal damage and promoting motor function recovery in SCI mice [102]. Furthermore, some authors report suppressing inflammation by activating the PI3K/AKT pathway to promote microglial M2 polarization or inhibit M1 polarization [97, 98]. Zhu et al. reported that Mg/Al layered double hydroxide NPs could increase transforming growth factor-β receptor 2 (TGFBR2) gene expression, leading to promotion of microglia/macrophage polarization from M1 to M2 [103]. The mechanism of action via which nanomaterials inhibit inflammation is complex and requires further in-depth research.

## Nanomaterials as therapeutic agents for management of dynamic spatiotemporal inflammation

Although early surgery and MP therapy are widely accepted modes of SCI treatment, the effectiveness of surgery is limited, and MP can cause complications, such as gastrointestinal bleeding, septicemia, and pulmonary embolism. Therefore, there is considerable debate surrounding the treatment options for SCI. Current fundamental research on SCI treatments has incorporated stem cells and genetically modified cell transplants, growth factors, biomolecules, and drug delivery systems in the search for effective treatment modalities. However, many of these agents have not exhibited appropriate efficacy in clinical trials [17]. This might be due in part to the treatment strategies being incompatible with the pathological process associated with dynamic spatiotemporal inflammation. Additionally, limited pharmacological efficacy may result from a low drug concentration at the injured site following administration via conventional routes or the development of potentially unacceptable side effects. To overcome these limitations, an urgent need exists for the development of treatments to counteract the progression of dynamic spatiotemporal inflammation; hence, a multi-target therapeutic approach might be promising for patients with SCI.

Nanomaterials boast several advantages, including the ability to cross the BSCB, act as drug carriers, accumulate and release drugs at the lesion site, have fewer adverse effects, enable responsive or triggered release, and support combination therapy [104–107]. Current immunomodulatory nanomaterials enhance SCI treatment efficacy through targeted delivery, responsive release, and improved biocompatibility. Such nanomaterials can be engineered to target specific inflammatory cells, such as microglia, neutrophils, and astrocytes, employing strategies such as encapsulation, stabilization, controlled release, and surface engineering to maintain the bioactivity of immune agents during circulation. Encapsulation protects therapeutic agents from enzymatic degradation, stabilization maintains their structural integrity, controlled release ensures gradual and consistent delivery, and surface modifications like PEGylation extend circulation time [108], all of which contribute to maximizing the therapeutic efficacy while minimizing systemic exposure [109]. Thus, considering their broad-spectrum efficacy, nanomaterials have been investigated extensively in different SCI models. These nanomaterials are categorized into distinct groups based on their source composition, including organic nanomaterials, bioderived nanomaterials, inorganic nanomaterials, and other nanomaterials.

### Organic nanomaterials

Organic nanomaterials primarily comprise polymer nanomaterials [110]. The main polymer materials currently used in tissue engineering for SCI are polylactic-co-glycolic acid (PLGA), hyaluronic acid (HA), poly DL-lactide-co-glycolide (PLG), and polyethylene glycol (PEG). Due to their inherent lack of imaging capabilities and native biocompatibility, extensive synthetic efforts are required to mitigate the need for additional processing steps and minimize cytotoxicity; these efforts involve techniques such as functionalization and surface modification [108]. Compared with inorganic nanomaterials, organic nanomaterials have better biodegradation and biocompatibility properties, making them suitable for extensive application as drug carriers.

The polymer nanomaterials loaded with anti-inflammatory drugs can reduce side effects, inhibit glial cell activation, and reduce inflammatory expression [111]. By exploiting the phagocytotic activity of macrophages and microglia during inflammation, NPs can act as a “Trojan horse” and be phagocytosed by macrophages to achieve the purpose of targeted delivery [112]. Polymeric NPs carrying anti-inflammatory drugs, such as minocycline, have been used to target and modulate M1-type macrophages through macrophage phagocytosis, promote macrophage polarization, and release anti-inflammatory drugs *in situ.* [113] In this manner, the dose of the drug can be adjusted, side effects can be reduced, and bioavailability and therapeutic effects can be improved. PLGA, polyethylenimine (PEI), and methoxyPEG (mPEG) are utilized to synthesize PLGA–PEI–mPEG (PPP) nanocarriers, which can support the anti-inflammatory drug etanercept (ET) and are modified with activatable cell penetrating peptide (ACPP)-targeting peptides, enabling their penetration of the blood–brain barrier to target the SCI site [114]. Nano drugs can effectively modulate polarization toward M2 macrophages, promote an anti-inflammatory immune response, and effectively treat SCI. In addition, due to the heightened expression of matrix metalloproteinases (MMPs) at the SCI site, the MMP-responsive molecule ACPP is incorporated into the biocompatible polymer PPP, enabling the NPs to specifically target diseased tissue. Meanwhile, the clinical anti-inflammatory drug ET can be loaded onto the polymer to modulate macrophage polarization and promote motor recovery. This organic polymer nanosystem exhibits effective lesion targeting and can inhibit the production of pro-inflammatory cytokines while promoting the production of anti-inflammatory cytokines, reducing the proportion of M1 macrophages and increasing the proportion of M2 macrophages [115]. After using these NPs, a significant decrease in the expression of M1-type markers can be observed at the injury site 3 dpi, indicating that the proportion of M1-type macrophages can be reduced within three days. HA can reportedly inhibit astrocyte proliferation and activation in the CNS by interacting with CD44 receptors to downregulate inflammatory signaling pathways [116]. Therefore, many nanomaterials use HA as a targeting molecule.

**Fig. 4:**
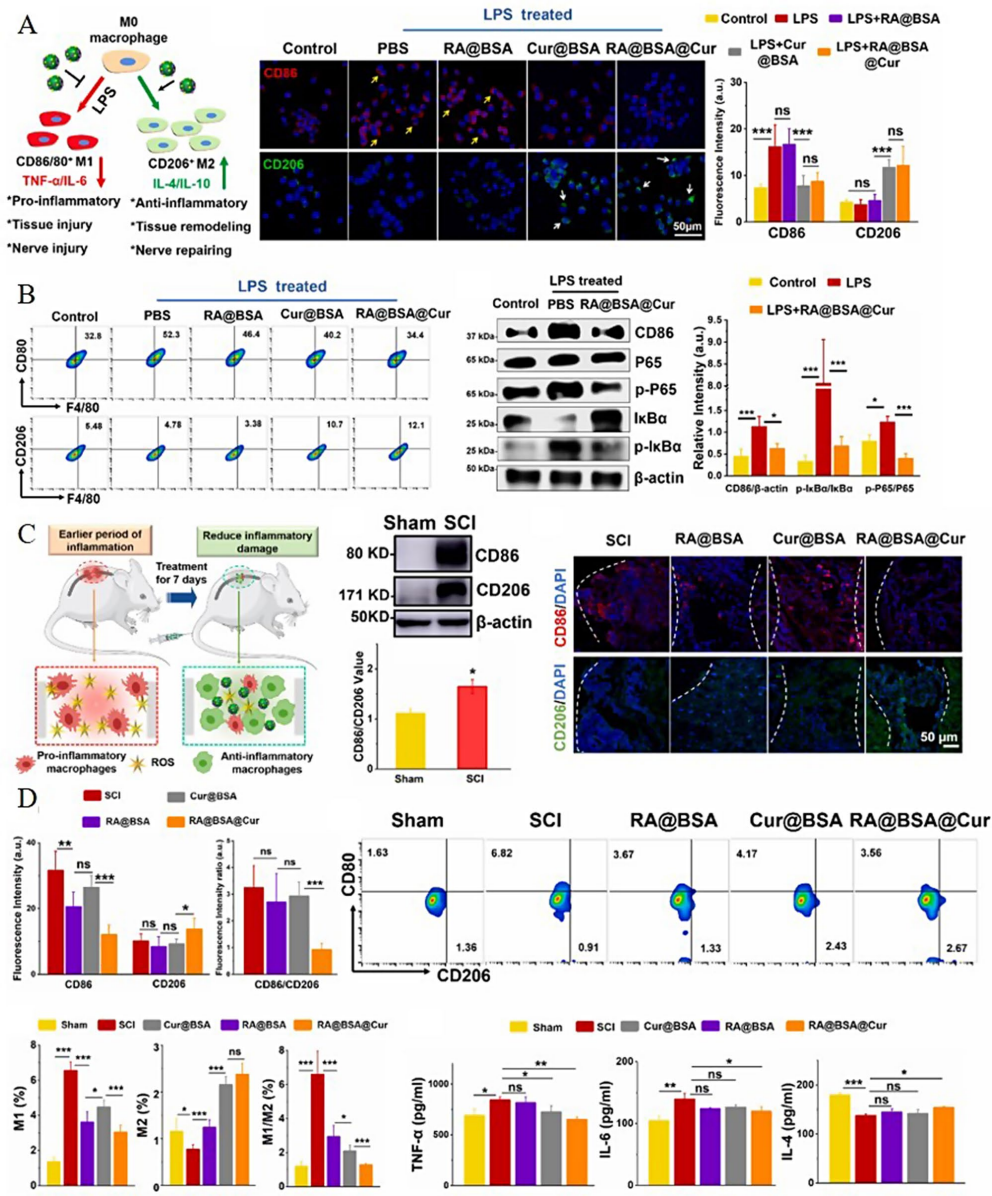
Therapeutic outcomes of RA@BSA@Cur NPs. **A**) Immunofluorescence and quantification of RAW264.7 cells cultured with different BSA-related nanoparticles (NPs) in the presence of lipopolysaccharide (LPS); **B**) Flow cytometry analysis and quantification of RAW264.7 cells cultured with various BSA-related NPs in the presence of LPS. The inhibition of the M1 phenotype and promotion of the M2 phenotype regulated by RA@BSA@Cur NPs may involve the NF-κB pathway. **C**) Changes in the expression of CD86 and CD206 under normal conditions and in the context of SCI. **D**) Ratio of CD80 + to CD206 + cells in different groups. Reproduced with permission [120]. Licensed under CC BY 4.0

Polymer-functionalized NPs are widely used in the medical field owing to their antioxidant and anti-inflammatory properties. For example, chitosan NPs can effectively improve the conduction of nerve impulses to the brain after SCI and can play an important role in the intervention of acute SCI [117]. Nano micelles (PSMs) have been prepared using polysialic acid (PSA) as a carrier and loaded with minocyclic acid (MC) to treat SCI. PSA can promote axon remodeling and tissue regeneration, while MC has an anti-inflammatory effect. Therefore, PSMs possess anti-inflammatory and neuroprotective properties, protecting damaged neurons and reducing scar formation, exhibiting a therapeutic effect [118]. Liu et al. prepared nano-antagonist hydrogel that mitigates inflammatory responses by specific calcium ion adsorption. In a contusive SCI mouse model, it successfully improved locomotor function [119]. Moreover, retinoic acid (RA) and curcumin (Cur) have been co-loaded with bovine serum albumin (BSA) to synthesize RA@BSA@Cur NPs (Fig. 4A). Cur has antioxidant properties and can effectively resist the ROS released during SCI. Meanwhile, RA can promote the regeneration and differentiation of axons, while albumin has favorable drug-loading properties and water solubility and is non-toxic. The constructed NPs harness the beneficial effects of both drugs and can be targeted to induce polarization towards M2-type macrophages, inhibit the release of inflammatory mediators, and reduce the inflammatory response (Fig. 4B-D) [120]. Owing to the dynamic spatiotemporal changes in inflammation following SCI, and targeting the increasing and peaking population of macrophages post-injury, administration of 150 µl of RA@BSA@Cur NPs at a concentration of 2 mg/ml via tail vein injection was selected 3 days after SCI. An increase in M2 macrophage polarization and a reduction in inflammatory effects were observed 7 dpi [120]. Notably, the therapeutic efficacy of these NPs in treating SCI can be attributed to both the intrinsic properties of the nanomaterials and the pharmacological effects of the incorporated drugs. The BSA matrix provides a biocompatible and stable platform for RA and Cur delivery, ensuring that the therapeutic agents reach the site of injury in a controlled and sustained manner. The synergistic action of RA promoting axon regeneration and Cur scavenging ROS, coupled with the targeted delivery mechanism of the NPs, contributes to the observed reduction in inflammation and enhancement of the regenerative microenvironment post-SCI. Therefore, the effectiveness of RA@BSA@Cur NPs in treating SCI is a result of the interplay between nanomaterial design and the therapeutic properties of the loaded drugs [120]. 

Immunomodulatory NPs carrying plasmid DNA or microRNA have been synthesized for delivery of nucleic acids to regulate macrophage polarization. For example, various HA–PEI NPs coated with IL-4/IL-10-expressing plasmid DNA or microRNA-223 can regulate macrophage polarization from the M1 to M2 phenotype [110]. Indeed, certain NPs can inhibit the inflammation-associated migration of neutrophils. Park et al. found that intravenous PLG NPs can be internalized by circulating neutrophils, leading to the reprogramming of neutrophil-related immune responses and reduced neutrophil abundance at the injury site. Consequently, the occurrence of fibrosis and glial scarring is reduced, and a microenvironment that promotes regeneration and functional recovery is maintained [110]. 

**Fig. 5:**
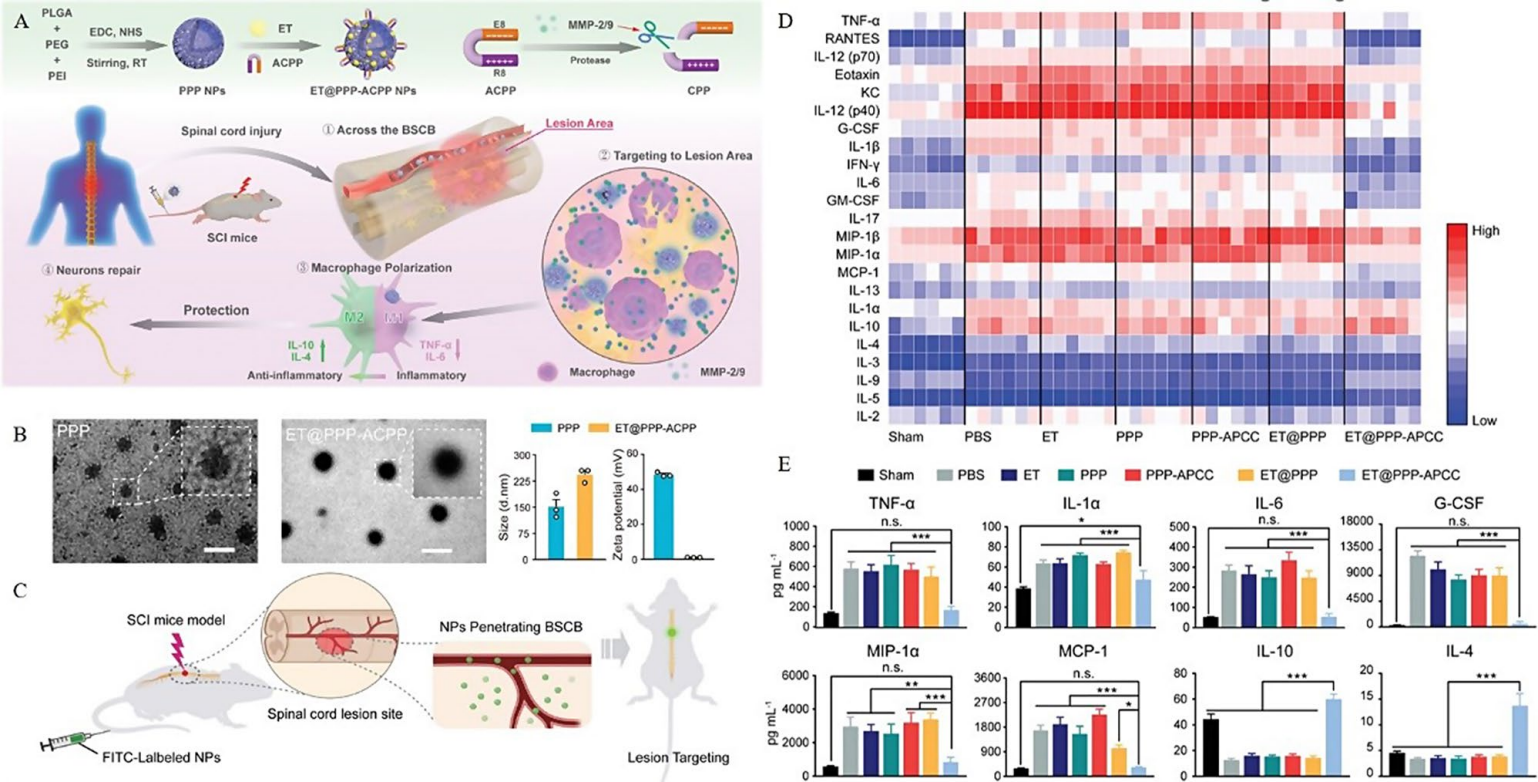
Therapeutic outcomes of ET@PPP-ACPP. **A**) Preparation of PPP and ET@PPP-ACPP. **B**) Characterization of ET@PPP-ACPP. **C**) Spinal cord injury (SCI)-targeting effect of PPP-ACPP NPs. **D**) Proinflammatory cytokine levels in the spinal cords of SCI mice, analyzed using the Luminex analysis system 24 h post-SCI. **E**) Levels of proinflammatory and anti-inflammatory cytokines in the spinal cord post-treatment. Reproduced with permission [115]. Copyright © 2023 Wiley

Dendritic macromolecules are highly branched, spherical, and low-dispersion synthetic molecules. Owing to their precise and controllable structure, dendritic macromolecules have an excellent drug-loading ability as carriers. Water-soluble NPs (PAMAM) and carboxymethyl chitosan (CMCht) have been used to synthesize CMCht/PAMAM dendrite NPs, which can effectively transport biological agents to the SCI site through endocytosis. These are readily internalized by glia when loaded with MP, which is released in the cells to regulate glial activity, inhibit the activation of astrocytes and formation of inflammatory factors, and promote axon regeneration. In fact, MP can be released continuously for up to 14 days [121]. In PPP–ACPP NPs, ACPP peptides target SCI sites. Leveraging the encapsulated etanercept, the NPs effectively home in on the lesion, balance the M1/M2 macrophage ratio, suppress inflammation, and accumulate prominently in the injured area, significantly enhancing the therapeutic efficacy for SCI (Fig. 5A-E) [115]. In addition, organic molecules or drugs can be combined with nanocarriers to achieve transport and anti-inflammatory effects. For instance, zein, which is amphiphilic, can penetrate the cell membrane and has excellent stability [122]. Meanwhile, aucubin—a member of the iridoid glycoside family—has anti-inflammatory and antioxidant effects; it can regulate the M1/M2 ratio of microglia/macrophages and reduce inflammation by blocking the NF-κB pathway [89]. 

### Bioderived nanomaterials

The term “bioderived nanomaterials” refers to nanoscale materials derived directly from living organisms or obtained by mimicking biological systems, including biomacromolecule nanomaterials (proteins and nucleic acids), extracellular vesicles (exosomes), and biological self-assembly systems (liposomes). These nanomaterials typically exhibit good biocompatibility, biodegradability, and specific biological activity; these properties allow their extensive utilization in biomedical and other fields.

#### Exosomes and cell-derived nanovesicles

Exosomes are nano-liposomes with a diameter of approximately 20–150 nm. They have paracrine functions and can quickly make corresponding physiological responses to help tissues recover from damage [123]. As extracellular vesicles, exosomes play a crucial role in intercellular communication and cargo transport.Exosomes exhibit a strong targeting ability, high safety, and low immunogenicity [124–126]. In addition, as drug carriers, they can extend the drug cycle time, improve bioavailability, and exhibit targeting properties [127]. Guo et al. developed intranasally administered mesenchymal stem cell-derived exosomes loaded with phosphatase and tensin homolog small interfering RNA (ExoPTEN) that migrated to the injured spinal cord area, attenuated PTEN expression, reduced microgliosis and astrogliosis, and significantly promoted functional recovery in rats with complete SCI [128]. Liu et al. prepared exosomes derived from iPSC-NSCs (iPSC-NSCs-Exos), which, in an SCI mouse model, were administered at different time points post-injury, targeting the expression levels of related proteins, and due to the peak content of microglia/macrophages occurring 7 dpi, the administration was set for 7 dpi. These exosomes can target microglia/macrophages and regulate LRIG3 expression by delivering let-7b-5p, thereby alleviating inflammation and enhancing motor function recovery. Seven days post-injury, a significant reduction in the expression of IL-1β and IL-18 was observed, indicating that the treatment based on the dynamic inflammatory process produced favorable results [129]. 

Mesenchymal stem cells (MSCs) have angiogenic, anti-apoptotic, and immunomodulatory effects. MSCs are driven to the injury site, where they promote polarization and reduce inflammation [130]. The therapeutic effects of MSCs are derived primarily from paracrine mechanisms. MSC-derived exosomes can downregulate pro-inflammatory cytokine levels and upregulate anti-inflammatory cytokine levels, reduce inflammation, and promote angiogenesis [131]. However, exosomes have unique advantages over MSCs in terms of manufacturing, storage, and application. Moreover, exosomes produced by M2 macrophages promote polarization of the M2 phenotype microglia/macrophages via a mechanism that may involve miRNA–mRNA interactions [132]. circRNA (circRNA/circ) is a type of non-coding RNA with a regulatory role in neural and inflammatory processes that is abundant in exosomes [133]. Bone marrow MSC exosomes can inhibit inflammation and treat SCI by secreting circRNAs. However, miRNA can be combined with exosomes to create, for example, exosomes rich in miR-124-3p. Exosomes can also be used as carriers to deliver miRNA to the microglia and inhibit the activation of M1 microglia and astrocytes, slow down the progression of inflammation after SCI, and aid recovery [134]. 

As emerging drug carriers, extracellular vesicles are characterized by high biocompatibility and low cytotoxicity and thus have a wide range of drug delivery prospects. The extracellular vesicles derived from MSCs are extracellular nanovesicles with the properties of MSCs and the specifications of NPs that can cross the cerebrospinal barrier to elicit antioxidant and anti-inflammatory effects in SCI. Extracellular vesicles from M2 macrophages can inherit various anti-inflammatory cytokines. Hence, due to the drug delivery advantages and anti-inflammatory effect, extracellular vesicles can be used as ideal drug carriers for treating SCI-associated inflammation [135]. Indeed, its effective combination with drugs to treat SCI is bound to elicit a 1 + 1 > 2 effect, affording it broad future prospects. Xiong et al. explored the use of extracellular vesicles (EVs) derived from mouse umbilical cord MSCs (MUMSCs) loaded with curcumin (Cur-EVs) for treating traumatic SCI. They incubated MUMSCs with curcumin for 48 h, and then isolated both regular EVs and Cur-EVs using ultracentrifugation. In a mouse model of complete SCI, Cur-EVs/EV administration significantly improved locomotor function and reduced inflammation compared to untreated model mice. Notably, Cur-EVs demonstrated superior effectiveness over regular EVs in enhancing the structural and functional recovery of SCI, including promoting macrophage polarization from the M1 to M2 phenotype and improving axonal regeneration in the lesioned areas [136]. 

**Fig. 6:**
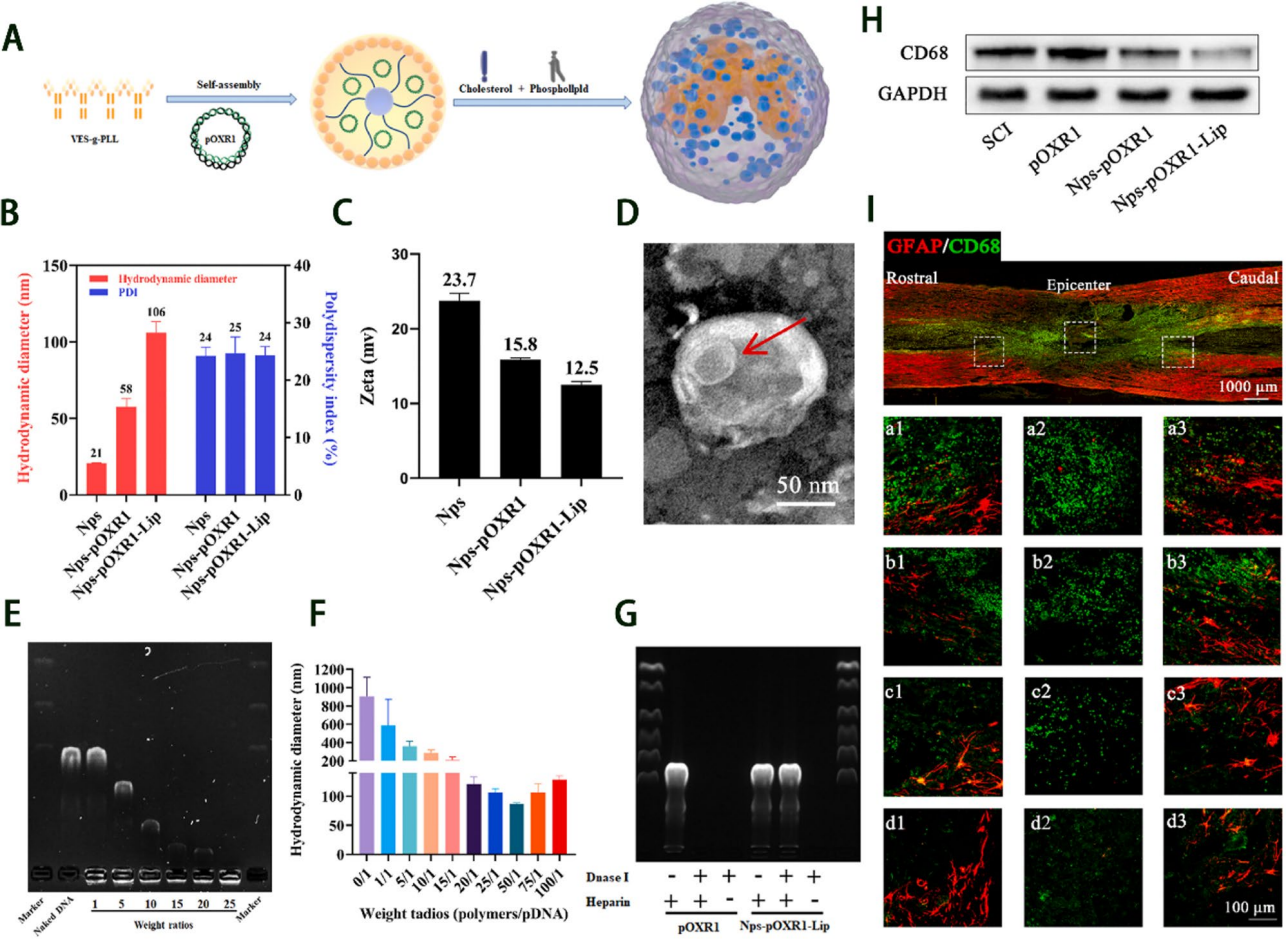
Therapeutic outcomes of NPs-pOXR1-Lip. **A**) Characterization of NPs-pOXR1-Lip. **B**-**D**) NPs-pOXR1-Lip decreases apoptosis following spinal cord injury. (SCI). **E**–**G**) NPs-pOXR1-Lip diminishes fibrotic scar tissue by influencing the inflammatory response. **H**, **I**) NPs-pOXR1-Lip facilitates functional recovery post-SCI. Reproduced from with permission [137]. Licensed under CC BY 4.0

A newly developed vector (i.e., pOXR1) can effectively deliver OXR1 plasmids (Fig. 6A). Meanwhile, a vitamin E succinate-grafted ε-polylysine (VES-g-PLL) polymer has been successfully utilized to create cationic NPs.The process of pre-compressing pOXR1 with NPs and then encapsulating it in cationic liposomes can help achieve a high encapsulation efficiency, good stability, and improved transfection efficiency. Indeed, NPs-pOXR1-Lip can prevent the degradation of DNA by DNase I, preserve DNA activity, and promote functional recovery by alleviating oxidative stress and suppressing inflammation (Fig. 6B-I) [137]. 

#### Membrane-coated nanomaterials

Membrane-coated NPs comprise a new type of nanomaterial consisting of cell biofilm-coated nanomaterial, typically synthesized via extrusion and ultrasonography. As they combine the cell membrane with nanomaterials, they incorporate the biological activity of cells and the therapeutic effect of nanomaterials. Currently, these NPs are widely used in the targeted delivery of drugs. Moreover, due to the antigen profile inherited from the source cell, membrane-coated NPs can act as decoys to neutralize and absorb diverse and complex pathological molecules, creating an anti-inflammatory microenvironment [110]. Neutrophil decoys (NDs) have been created with biomimetic properties similar to those of neutrophils in regulating inflammation and clearing ROS to regulate the microenvironment [138]. They can also adsorb inflammatory factors, reduce the overactivation of macrophages, microglia, and astrocytes, and delay the progression of inflammation [138]. Thus, by taking advantage of the sharp increase in the levels of TNF-α, IL-1β, and IL-6 in the spinal cord and serum within 6 h post-SCI, NDs can be targeted to the spinal injury site to address the rapidly amplifying inflammatory response during the early stage of SCI [138]. 

**Fig. 7:**
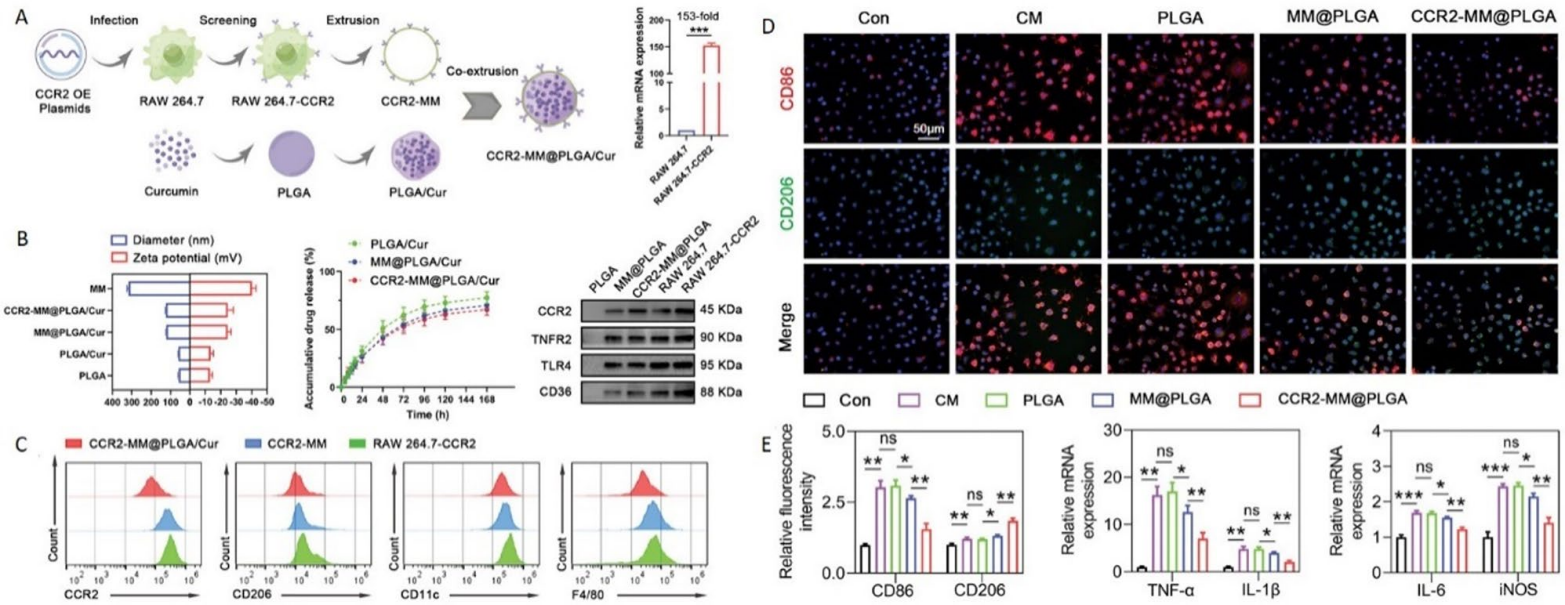
Preparation and therapeutic effects of CCR2-targeted PLGA/curcumin nanoparticles (CCR2-MM@PLGA/Cur/NPs). **A**) Preparation of CCR2-MM@PLGA/Cur/NPs. **B**) Average diameters and zeta potentials. In vitro drug release characteristics of PLGA/Cur, MM@PLGA/Cur, and CCR2-MM@PLGA/Cur. Results from western blot analyses showing the expression levels of CCR2, TNFR2, TLR4, and CD36 in PLGA/NPs, MM@PLGA/NPs, CCR2-MM@PLGA/NPs, RAW 264.7, and RAW 264.7-CCR2 cell lines. **C**) Analysis via FCM of CCR2 and specific protein biomarkers (CD206, CD11c, and F4/80) expression in CCR2-MM@PLGA/Cur, CCR2-MM, and RAW 264.7-CCR2 cells. **D**) Immunostaining representative images showing CD86 (in red) and CD206 (in green) in BV2 cells across various experimental groups. **E**) Quantitative immunofluorescence of CD86 (indicating pro-inflammatory) and CD206 (indicating anti-inflammatory) in microglia, and the relative expression of pro-inflammatory genes (TNF-α, IL-1β, IL-6, and iNOS), normalized to control levels [140]. Reproduced with permission. Copyright © 2023 Wiley

An et al. developed macrophage membrane bionic-modified nanoliposomes (MH-DS@M-Lips) loaded with minocycline hydrochloride (MH) and dextran sulfate (DS) to target the injury site and alleviate secondary injury in SCI. These nanoliposomes are designed to bind calcium ions in situ, forming metal ion complexes that lower calcium ion concentrations, while also slowly releasing MH to enhance anti-inflammatory effects. Considering the elevation of macrophages occurs within 7 dpi, they selected the 7-day period post-injury as the sustained injection window, during which the slow release of MH promotes the transformation of macrophages from the M1 to the M2 phenotype, targeting the treatment of inflammation [139]. Gu et al. prepared a multifunctional biomimetic nano platform specifically designed for the CCL2–CCR2 axis (CCR2-MM@PLGA/Cur); the platform consists of an engineered macrophage membrane (MM) coating with elevated CCR2 expression and PLGA NPs encapsulating therapeutic drugs (Fig. 7A-C). Overexpression of CCR2 on the MM enhances targeted drug delivery to the injury site and reduces macrophage infiltration, pro-inflammatory polarization of microglia, and neuronal apoptosis by facilitating the capture of CCR2 ligands. Owing to CCR2 effectively targeting CCL2 secreted by microglia according to the dynamic spatiotemporal changes of inflammation, NPs are internalized by macrophages on the third day after injection, and the level of various pro-inflammatory factors is reduced seven days later. With the dynamic changes in the microglial population, fluorescent signals can be detected as early as one day after injection, peaking at three days, which helps to inhibit the progression of early inflammatory cascades following SCI. After injection, the NPs are internalized by macrophages on the third day and a decrease in various pro-inflammatory factors can be detected seven days later (Fig. 7D-E) [140]. 

### Inorganic nanomaterials

Inorganic nanomaterials include silica NPs, metal NPs, carbon nanotubes, quantum dots, among others. Their preparation process is simpler than that for organic NPs, which typically require linking or coating with a “biocompatible” coating [108]. However, due to the different preparation materials and contents, the presence of heavy metals can result in cytotoxicity, making it necessary to monitor their safety profiles carefully.

Metal nanoclusters are smaller (< 3 nm) than traditional nanomaterials and are advantageous owing to their biocompatibility, low cytotoxicity, high cleaning efficiency, and blood–brain barrier-crossing ability. For example, gold NPs (AuNPs) are gradually being developed for potential use in biomedicine owing to certain anti-inflammatory properties. AuNPs, which are small and exhibit surface plasmonic resonance and quantum tunneling effects, can strongly bind to terminal sulfhydryl groups (-SH), and they contain negatively charged ions. Therefore, they can serve as drug carriers to ensure the controlled delivery of drugs to the target location [141, 142]. Moreover, they reportedly reduce the number of M1 macrophages while increasing that of M2 macrophages in the spleens of septicemic mice [110]. Lin et al. developed zinc-modified R-DHLA-stabilized gold nanoclusters that promoted the polarization of pro-inflammatory macrophages, reduced neuronal ROS-induced apoptosis and inflammation, decreased lesion size, and increased the survival rate of ventral neurons in SCI therapy [143]. 

Silica NPs, common inorganic nanomaterials, are easy to synthesize. Owing to their size, shape, porosity, and other physical properties, silica NPs can be modified easily and thus are suitable for use in different applications. Silica NPs have unique advantages as drug carriers owing to their structure, large surface area, and nano-size, enabling their penetration of the blood–brain barrier and target areas of SCI [106]. Mesoporous silica NPs can be used to synthesize various drugs by leveraging porous networks according to different requirements. They can extend the circulation time of the loaded therapeutic agents, thereby increasing their half-life and reducing the overall concentration to minimize side effects [108]. A mesoporous silica NP carrier was used to deliver pirfenidone for managing inflammation after SCI. This carrier addressed issues related to the short half-life of pirfenidone, substantially reducing side effects throughout the treatment course and crossing the blood-brain barrier to reach the lesion sites with improved therapeutic efficacy [144]. Ma et al. developed polyethylenimine-conjugated, diselenide-bridged mesoporous silica NPs (MSN) to deliver siRNA-IRF5, which regulated M1-to-M2 macrophage polarization by silencing IRF5, thereby suppressing excessive inflammation, enhancing neuroprotection, and promoting locomotor restoration in a crush SCI mouse model [145]. 

Graphite has conductive properties that can mediate signal transduction between cells and promote trauma recovery. Functional graphene nanomaterials have high load capacity and adhesion ability, which facilitate effective targeting of select areas and release of drugs over long periods for therapeutic purposes [146]. Graphene quantum dots are 1–10-layer-thick sheets of graphene, a carbon allotrope comprising a single layer of carbon atoms in a honeycomb structure. They hold great potential for use as bioimaging agents and biosensors owing to their small size, high surface–volume ratio, excellent electrical properties, and low toxicity [147]. Selenium is an essential trace element that strengthens the immune system and protects against free radical-induced damage [148]. While the safe dose range of selenium is very narrow, synthesized selenium NPs exhibit low toxicity, high antioxidant activity, and immune regulatory properties. Therefore, selenium nanomaterials have become a research hotspot. In particular, they have good anti-inflammatory properties, which are conducive to tissue regeneration after SCI [105]. The therapeutic effect of selenium NPs is related to particle diameter and dose. Moreover, selenium NPs are relatively safe at diameters of 25–80 and 50–250 nm. When the diameter is < 200 nm, the specific surface area provides more active sites, and the free radical-scavenging efficiency is increased significantly [148]. Selenium-doped carbon quantum dots (Se-CQDs) have been ingeniously synthesized, exhibiting remarkable anti-inflammatory properties. These Se-CQDs actively mitigate inflammation in the affected tissues, safeguarding cells from inflammatory damage, particularly under conditions such as SCI-associated neuroinflammation. By suppressing the activation of pro-inflammatory cytokines and modulating the immune response, they significantly hinder the progression of inflammatory cascades [104]. Similarly, epigallocatechin-3-gallate selenium NPs can effectively remove ROS from lesion sites and reduce inflammation [149]. Zhou et al. developed a synergistic therapeutic strategy using multifunctional selenium NPs. These NPs promote mitochondrial homeostasis, regulate inflammatory factor expression, and facilitate the polarization of macrophages to an M2 phenotype [150]. Luo et al. prepared CD44-targeting hyaluronic acid-selenium (HA-Se) NPs that effectively scavenged ROS, suppressed inflammatory responses by mitigating the secretion of proinflammatory cytokines, and enhanced functional recovery in a rat model of SCI [151]. 

CeNPs are composed primarily of CeO_2_ and exhibit remarkable antioxidant, anti-inflammatory, and anti-apoptotic effects. As the surface of these NPs contains several Ce^3+^/Ce^4+^ REDOX active sites, they have a high antioxidant capacity. In the acute phase following SCI, they can inhibit inflammation, while in the subacute stage, they reduce the damage cavity and pro-inflammatory cytokine expression and increase anti-inflammatory factor expression. Subsequent significant improvement in motor function has been reported in the chronic stage. Therefore, CeO_2_ can reduce ROS levels after SCI, as well as the level of inflammation at the injury site, and promote repair after inflammation [105]. Inflammatory cells, when treated with CONPs in vitro, have been found to markedly reduce their expression of acute inflammatory and apoptosis-regulating molecules, including Cox2, Nr-f2, P53, Casp3, IL-1β, IL-6, and TNF-α, according to a pioneering study. CONPs can induce the polarization of M1 macrophages to M2 macrophages, thereby promoting axonal regeneration. In the dynamic changes following SCI, the peak in macrophage numbers occurs on the fifth day; therefore, drug administration is scheduled at this time point to achieve optimal therapeutic effects. A decrease in levels of iNOS, IL-1β, IL-6, and TNF-α can be detected within the first week of treatment, indicating good anti-inflammatory effects. However, this improvement has an optimal therapeutic dose range (500–1000 µg mL^-1^), beyond which CONPs can have adverse effects on functional recovery. Therefore, administering appropriate doses of local CONPs is crucial for enhancing the efficacy of any drug, gene, or cell therapy for acute SCI [152]. A previous study developed a smart carrier that incorporates cerium oxide NPs with enzyme-mimicking properties. Cerium oxide NPs restore the responsiveness of pro-inflammatory M1 macrophages to calcitonin gene-related peptide (CGRP) by upregulating RAMP1, shifting macrophages toward an anti-inflammatory M2 phenotype. The cerium oxide NPs improved hindlimb locomotor function significantly in SCI rats. This work highlights the potential of cerium oxide NPs as a promising therapeutic candidate for SCI and other conditions characterized by similar inflammatory states [153]. 

As a two-dimensional (2D) transition metal disulfide compound nanomaterial, MoS_2_ has good conductivity, catalytic activity, biocompatibility, and intercalable structure. Hence, MoS_2_ is currently being used in the development of drug delivery nanocallers [154]. MoS_2_ regulates the polarization of macrophages, promotes anti-inflammatory effects, and repairs and protects the nerves. Its high elasticity and low friction coefficient enable it to alleviate mechanical stress on the spinal cord and serve as a natural carrier. Researchers have synthesized polyvinyl alcohol (PVA) and MoS_2_ to create hydrogels, where PVA inhibits the migration of inflammatory cells and reduces secondary injury post-SCI, while MoS_2_ suppresses M1 and promotes M2 differentiation and activation. The synthesized hydrogels exhibit good flexibility, an appropriate Young’s modulus, and high electrical conductivity, which can facilitate the targeted differentiation of NSCs and scavenge ROS from the injury site [154]. Additionally, the MoS2@PEG nanoflowers created by incorporating PEG with MoS_2_ nanosheets act as carriers for anti-inflammatory drugs. The nanoflowers can extend the therapeutic time window to over 96 h and regulate the polarization of macrophages to elevate the levels of anti-inflammatory factors [155]. 

**Fig. 8:**
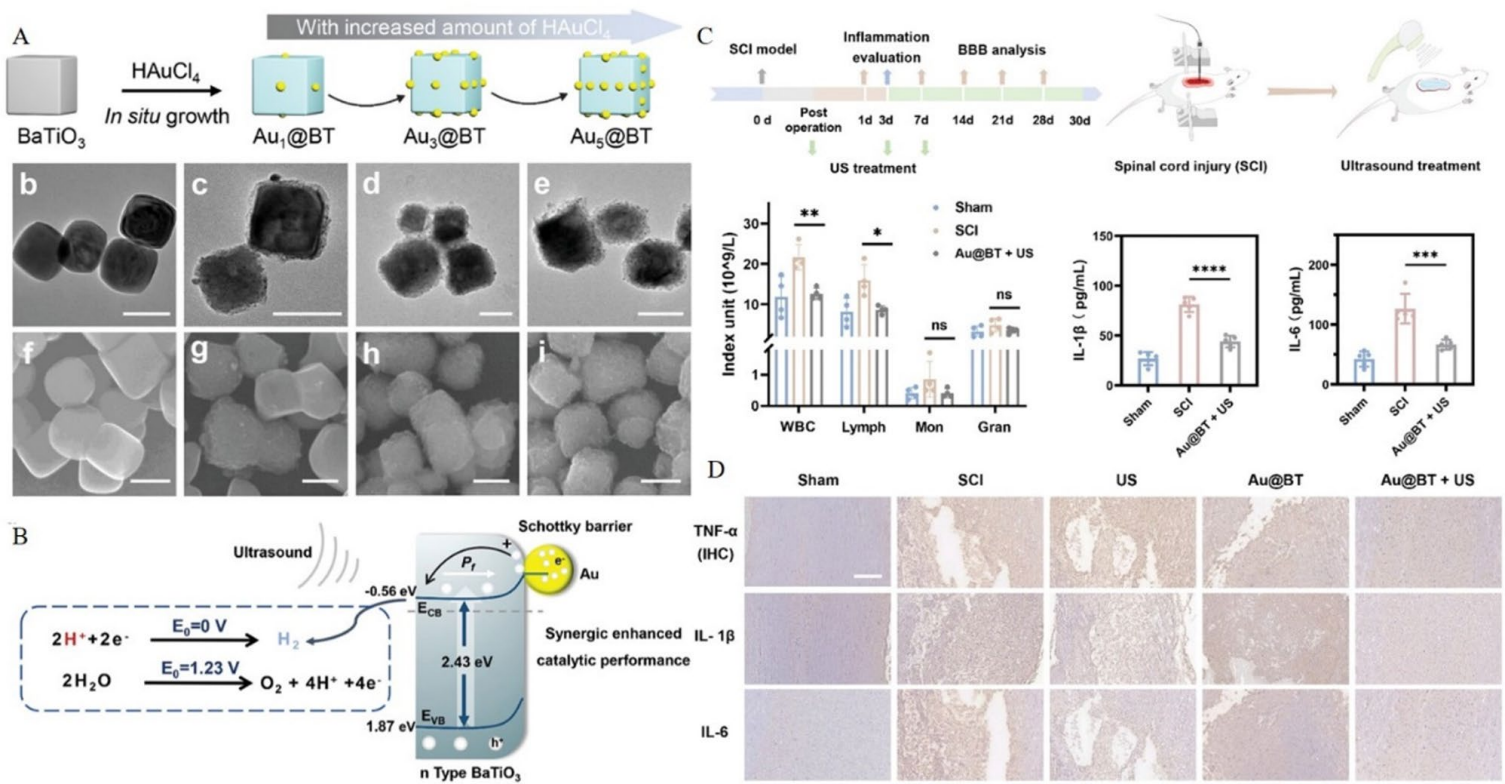
Au_x_@BT nanoparticles alleviate spinal cord injury by generating hydrogen. **A**) Au_x_@BT nanoparticle synthesis and characterization; X = 1, 3, or 5. **B**) Principle underlying the generation of H_2_. **C**) Illustration of the ultrasound-assisted piezoelectric catalytic therapy for rats with spinal cord injury. Numbers of inflammatory cells and levels of IL-1γ and IL-6 in the blood of rats. **D**) Representative histological images corresponding to the expression of TNF-α, IL-1γ, and IL-6. Reproduced with permission [158]. Copyright © 2023 Wiley

Zinc oxide NPs are relatively common and have low toxicity and good biocompatibility and bioactivity. These NPs can be applied locally to ameliorate the inflammatory microenvironment and reduce the dysfunction caused by secondary injury [156]. Moreover, a highly bioactive iridium complex (IrFPHtz) has shown strong antioxidant properties as well as neuroprotective and anti-inflammatory effects [157]. IrFPHtz can enhance the expression of superoxide dismutase (SOD)1—a key antioxidant enzyme—effectively mitigating ROS generation. Thus, it safeguards neurons and myelin sheath integrity against inflammation-induced damage, thereby inhibiting the formation of a dense glial scar. This therapeutic action helps preserve neural function, facilitates the creation of a conducive microenvironment for neuronal repair, and promotes the regrowth of elongated axons within the glial scar, contributing to neural tissue restoration following inflammatory insults [157]. Due to its selective antioxidant properties, ease of penetration, and good biosafety, hydrogen (H_2_) is widely studied as a potential anti-inflammatory gas for treating SCI. A particle nanocomposite material featuring a Schottky heterojunction was synthesized through the in-situ growth of AuNPs on piezoelectric BaTiO_3_ (Au@BT) (Fig. 8A-B). This composite material can generate H_2_ continuously by catalyzing H + reduction through piezoelectric catalysis and achieve anti-inflammatory effects by scavenging intracellular ROS. Furthermore, Au@BT NPs can act as potential neuroprotective agents. With the assistance of ultrasonography, these NPs can scavenge free radicals by producing H_2_ and further protect the nerves by regulating the tryptophan metabolism pathway (Fig. 8C-D) [158]. 

**Fig. 9:**
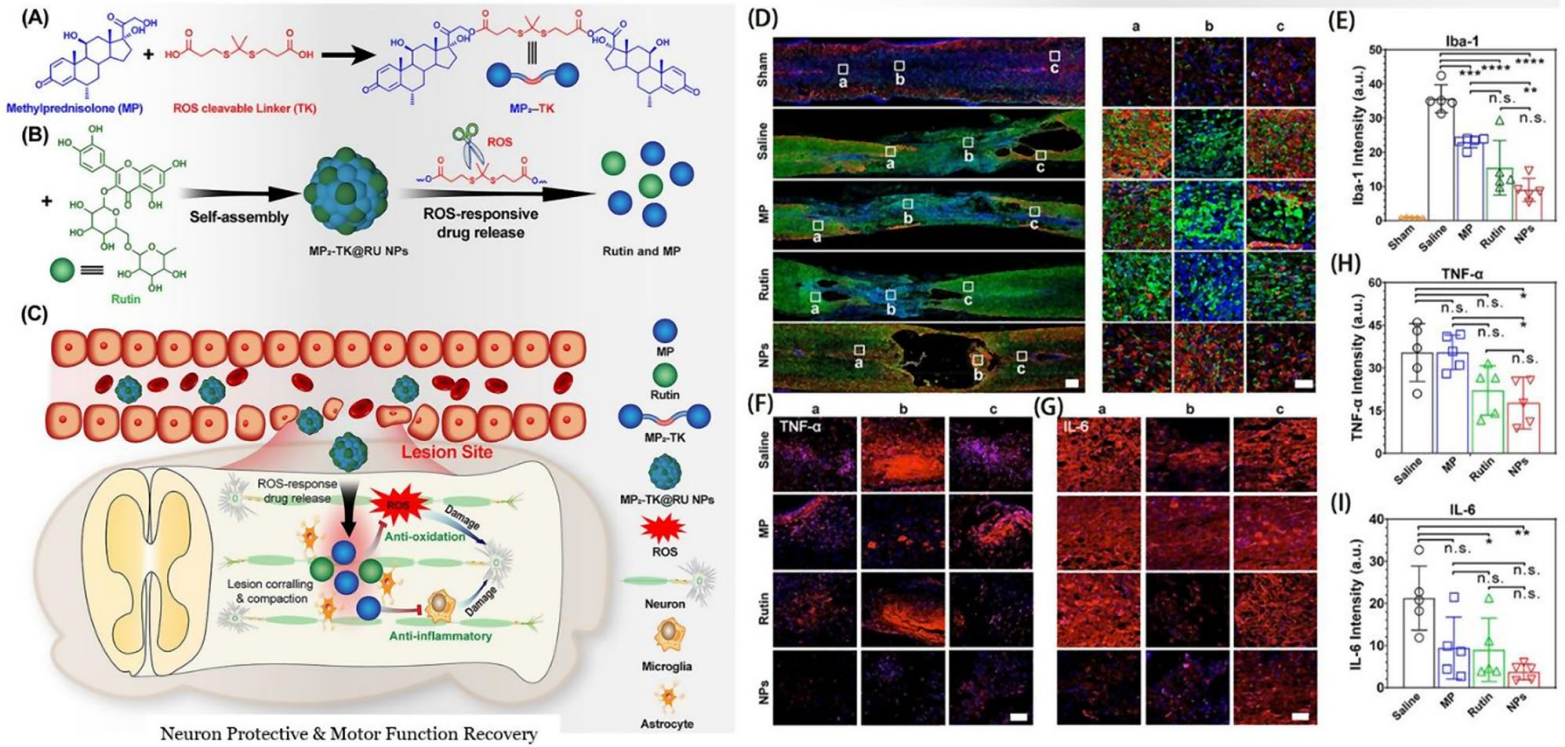
Assembly and therapeutic effects of MP2-TK@ RU nanoparticles. **A**) Synthesis of the precursor MP2-TK. **B**) Self-assembly and ROS-responsive behavior of MP2-TK@RU NPs. **C**) Treatment principles of spinal cord injury. **D**) Representative immunofluorescence images of injured spinal cords eight weeks post-different treatments. **E**) Relative intensity of Iba-1 immunofluorescence in injured spinal cords. **F**, **G**) Magnified immunofluorescence images of TNF-α. **H**, **I**) Relative intensity of TNF-α **H**) and IL-6 **I**) immunofluorescence in injured spinal cords. Reproduced with permission [107]. Copyright © 2023 American Chemical Society

Wang et al. developed carrier-free thioketone-linked MP dimer@rutin NPs (MP2-TK@RU NPs), achieving combined therapy for SCI by co-assembling MP dimers and rutin **(**Fig. 9A**)**. These compounds are designed to be cleavable by ROS (Fig. 9B). This nanomedicine offers several advantages: (1) the carrier-free system is straightforward to produce and has a high drug-loading capacity; (2) the ROS-cleavable linker enhances the efficiency of targeted drug delivery to the injury site; (3) rutin, a natural flavonoid derived from plants, possesses good biocompatibility along with anti-inflammatory and antioxidant properties. The combined application of nanomedicine and anti-inflammatory drugs can effectively enhance therapeutic efficacy. Indeed, MP2-TK@RU NPs demonstrate potent anti-inflammatory and antioxidant properties both in vitro and in vivo, along with neuroprotective effects and improvement in motor function recovery, and it can effectively mitigate the complications associated with MP use (Fig. 9C-I) [107]. The ROS-cleavable linkers enable the NPs to respond dynamically to the microenvironment at the injury site, where ROS levels are elevated. This dynamic response facilitates the localized release of rutin, maximizing its therapeutic impact while minimizing systemic exposure and side effects. Rutin’s inherent anti-inflammatory and antioxidant properties complement the ROS-scavenging capability of the NPs, creating a synergistic effect that enhances the overall therapeutic outcome. The high biocompatibility of rutin ensures that the NPs remain non-toxic to surrounding healthy tissue, which is particularly important given the delicate nature of the spinal cord environment. Moreover, the high drug-loading capacity of the carrier-free system ensures sustained release of rutin over time, which is beneficial for long-term management of SCI. Together, the design of the nanomaterial and the therapeutic effects of rutin provide a multifaceted approach to SCI treatment, addressing both immediate inflammatory responses and long-term regeneration and repair processes [107]. 

### Other nanomaterials

Tannic acid, as a crosslinking agent, has antioxidant, anti-inflammatory, and antibacterial properties; however, it cannot reach nerve tissue on its own [159]. Owing to their anti-inflammatory function, tannic acid-doped MSC extracellular vesicles have been developed for treating SCI and promoting nerve tissue repair. These vesicles can suppress the release of pro-inflammatory cytokines, including IL-6 and TNF-α. With the aid of carriers, these vesicles can be effectively released in the damaged site where they enhance the anti-inflammatory effects.

Low-dose estrogen can limit the activation of glial cells to improve motor function. There are studies on the surgical implantation of epidural gel patches to directly deliver NPs, which increase the therapeutic window of estrogen and effectively reduce the risk of venous thromboembolism caused by intravenous injection [160]. Moreover, methotrexate, a folate inhibitor with anti-proliferative and anti-inflammatory effects primarily used to manage inflammatory diseases and cancers, inhibits cell proliferation and interferes with the activation and migration of inflammatory cells (such as T cells, macrophages, and neutrophils). Nanomaterials can specifically target macrophages overexpressing the inflammation-activated folic acid receptor for anti-inflammatory therapy [110]. Additionally, butylphthalein elicits anti-inflammatory and protective effects by reducing the levels of pro-inflammatory molecules, ROS, and autophagy, promoting M2 polarization, and regulating T-cell function [161]. 

Veneruso et al. prepared a novel nanogel capable of selectively releasing active compounds in microglia and astrocytes. In the early stage, the nanogel loaded with rolipram (an anti-inflammatory drug) improves animal mobility significantly due to a greater number of microglia and macrophages localizing to the lesions. However, advanced treatments are not as effective, with poor motor recovery due to extensive degenerative changes. The results indicate that nanocarriers have selectivity and functionality in the context of targeting glial components at different stages of SCI, providing new treatment options for glia-mediated inflammation following neurodegenerative events in the CNS [162]. 

Fan et al. designed a novel nano complex for targeted drug delivery to treat SCI, featuring a mesoporous silica NP core loaded with microRNA and enveloped in a human umbilical cord MSC membrane modified with rabies virus glycoprotein (RVG). The design allows the nano complex to efficiently cross the compromised BSCB and accumulate at the injury site due to its exosome-like characteristics and the targeting ability of RVG. Upon accumulation, the nano complex releases microRNAs that stimulate axon growth and modulate the inflammatory microenvironment, promoting M2 polarization of microglia and reducing inflammation [163]. Xin et al. utilized a microfluidic chip to create imine-crosslinked aldehyde-methacrylate-hyaluronan/collagen hybrid hydrogel microfibers loaded with IL-4-containing ZIF-8 NPs (IL4@ZIF-8 NPs). These microfibers mimic the natural ECM, exhibit neuroinductive properties, and respond to the acidic microenvironment by releasing IL-4, which promotes the polarization of recruited macrophages toward an M2 phenotype and inhibits inflammation. When implanted into SCI rat models post-SCI, the content of macrophages, which normally peaks at seven days, was significantly reduced and polarized toward the M2 type [164]. 

## Conclusions and prospects

SCI-induced neuroinflammation exhibits complex spatiotemporal characteristics, with interconnected and interacting elements posing challenges to the precise characterization of the process. While single-cell sequencing has highlighted numerous cell subsets or subtypes that could serve as key therapeutic targets, confirmatory research remains scarce. Looking ahead, deciphering the intricate spatiotemporal mechanisms of neuroinflammation in SCI requires further in-depth studies. Moreover, the paucity of effective means to augment neural regeneration capacity and the persistent hindrance to substantial nerve regeneration constitute ongoing obstacles. Consequently, SCI continues to present a formidable challenge. Strategies aiming to modulate the dynamic spatiotemporal neuroinflammatory response in SCI have garnered significant research attention. However, reliance on a single intervention strategy is insufficient to efficaciously facilitate recovery from SCI, suggesting that future clinical interventions may require multifaceted synergistic approaches. This underscores the requirement for interdisciplinary collaboration within neuroscience to forge a holistic therapeutic strategy, integrating surgical interventions with biological rehabilitation modalities, thereby comprehensively addressing the multifarious nature of SCI.

The field of nanomaterial-based immunotherapy has seen substantial progress in recent decades, showcasing notable potential for clinical application. Through continuous advancements in manufacturing techniques and the implementation of sophisticated design concepts, nanotechnology has been effectively applied in the field of immunological regulation, enabling the management of a wide range of diseases [165]. Nevertheless, there has been insufficient research on the use of nanomaterials for modulating dynamic spatiotemporal inflammation, which has received growing recognition. Consequently, exploring the complex interplay between nanomaterials and dynamic spatiotemporal inflammation has become a novel area of research within nanomedicine and neurology [166]. A comprehensive understanding of the positive and negative aspects of each nanomaterial will facilitate their appropriate use and enable their development as effective modalities for managing dynamic spatiotemporal inflammation. If nanomaterials can be developed to counter the effects of different inflammatory cells in the background of SCI, they would be able to specifically target each cell subset, the counts and functions of which are dynamically altered during inflammation. Such targeted treatment measures can compensate for the disadvantage that, in the existing treatment modalities, the speed of drug therapy cannot match the dynamicity of inflammation.

Future research hotspots in the field of nanomaterials for addressing dynamic spatiotemporal inflammatory responses will likely involve the design of intelligent nanomaterials. These materials should be capable of dynamically or smartly regulating interactions between different cells within the inflammatory environment. For example, a system can be designed wherein drugs targeting different inflammatory cells can be released in a stepwise manner based on the dynamicity of inflammation in SCI to achieve better efficacy. Alternatively, slow-release drugs can be designed by exploiting the different dynamic phenomena observed during inflammation. In the early stage, inflammation itself can be used to prevent the spread of injury. That is, when the negative effects of inflammation gradually become prominent, inflammation can be controlled through drug activity. However, research on the dynamic modulation of inflammation in SCI using nanomaterials is relatively scarce. Organic NPs, which display good biocompatibility, represent a hotspot in nanomaterial research. Consequently, it is highly likely that these nanomaterials can facilitate swift bioengineering transformations within limited timeframes.

Upcoming research endeavors should focus on exploring and refining strategies for active targeting in nanomaterial design, with the primary goal of precisely enhancing drug accumulation at the lesion site and reducing side effects. This targeted approach ensures that the therapeutic agent is released only when and where it is needed, thereby minimizing systemic exposure and reducing the risk of adverse effects on non-target tissues. In addition, nanomaterials can be surface-functionalized with ligands that bind specifically to inflammatory cells, and they can incorporate responsive release mechanisms that trigger drug release in the presence of inflammation-related cues such as pH changes or presence of ROS. Third, using biocompatible and biodegradable materials reduces the risk of chronic toxicity. Localized delivery methods could further reduce exposure to healthy tissues.

Constraints related to the rapid, accurate, and consistent biofabrication of NPs impede their clinical application. Additionally, the inherent toxicity of inorganic nanomaterials represents a bottleneck to their practical utilization in biomedicine. Therefore, the development of more efficacious and less invasive nano-scale therapeutic strategies by incorporating bioactive materials, such as exosomes and cellular membranes, is important. Owing to their high compatibility with human cells and tissues, these nanomaterials are poised to gain increased prominence, revolutionizing medical interventions by mimicking nature’s own delivery systems and enhancing treatment outcomes while minimizing the adverse effects. Simultaneously optimizing drug delivery to precisely target specific cells or tissues enhances therapeutic efficacy while minimizing adverse reactions. This targeted approach involves integrating targeting ligands and optimizing release kinetics to achieve higher therapeutic outcomes with reduced off-target effects. Ultimately, comprehensive safety assessments should be conducted to evaluate the long-term effects of NPs in vivo, including their biodistribution, metabolism, and clearance, to ensure safety within the body. Such assessments are critical for establishing the safety profile of NPs and for guiding the development of dosing regimens. Developing more efficient and scalable methods for the production of NPs ensures consistency and reproducibility, which are essential for clinical translation. Such steps could pave the way for gradual clinical translation, enabling effective application in patients and facilitating transition from bench to bedside.

Overall, exploring therapies that use nanomaterials to spatiotemporally, dynamically manipulate inflammation is a highly attractive and promising research field. Through continuous advancements in manufacturing techniques and implementation of sophisticated design concepts, nanotechnology has been utilized effectively in the field of spatiotemporal dynamic regulation. This emphasizes the increasing necessity to develop a comprehensive understanding of the alterations in dynamic spatiotemporal inflammation in SCI. Hence, further investigations are necessary to explore “smarter nanomaterials” consistent with dynamic spatiotemporal inflammation to enhance therapeutic capabilities, while concurrently mitigating the detrimental effects. Biocompatibility and safety still hinder clinical translation. Hence, it is imperative to conduct a comprehensive study and assess the local and systemic toxicity of nanomaterials. Although numerous obstacles remain, we are optimistic about spatiotemporal dynamic immunomodulation.

## Data Availability

No datasets were generated or analysed during the current study.

## Abbreviations

ACPP: Activatable cell penetrating peptide
Au@BT: AuNPs on piezoelectric BaTiO3
AuNP: Gold nanoparticle
BSA: Bovine serum albumin
CNS: Central nervous system
Cur: Curcumin
ET: Etanercept
HA: Hyaluronic acid
IL: Interleukin
iNOS: Inducible nitric oxide synthase
IrFPHtz: Highly bioactive iridium complex
MC: Minocyclic acid
MM: Macrophage membrane
MMP: Matrix metalloproteinase
mPEG: Methoxypolyethylene glycol
MSC: Mesenchymal stem cell
NSC: Neural stem cell
NP: Nanoparticle
PEG: Polyethylene glycol
PEI: Polyethylenimine
PLG: Poly DL-lactide-co-glycolide
PLGA: Polylactic-co-glycolic acid
PPP: PLGA-PEI-mPEG
PSA: Polysialic acid
PSM: Nano micelle
RA: Retinoic acid
ROS: Reactive oxygen species
SCI: Spinal cord injury
Se-CQD: Selenium-doped carbon quantum dot
TNF: Tumor necrosis factor
